# Accounting for Errors in Low Coverage High-Throughput Sequencing Data When Constructing Genetic Maps Using Biparental Outcrossed Populations

**DOI:** 10.1534/genetics.117.300627

**Published:** 2018-02-27

**Authors:** Timothy P. Bilton, Matthew R. Schofield, Michael A. Black, David Chagné, Phillip L. Wilcox, Ken G. Dodds

**Affiliations:** * Department of Mathematics and Statistics, University of Otago, Dunedin 9054, New Zealand; † Invermay Agricultural Centre, AgResearch, Mosgiel 9053, New Zealand; ‡ Department of Biochemistry, University of Otago, Dunedin 9054, New Zealand; § Palmerston North Research Centre, New Zealand Institute for Plant & Food Research Limited (Plant & Food Research), Palmerston North 4442, New Zealand

**Keywords:** genetic linkage maps, map inflation, genotyping-by-sequencing, sequencing errors, hidden Markov model

## Abstract

Next-generation sequencing is an efficient method that allows for substantially more markers than previous technologies, providing opportunities for building high-density genetic linkage maps, which facilitate the development of nonmodel species’ genomic assemblies and the investigation of their genes. However, constructing genetic maps using data generated via high-throughput sequencing technology (*e.g.*, genotyping-by-sequencing) is complicated by the presence of sequencing errors and genotyping errors resulting from missing parental alleles due to low sequencing depth. If unaccounted for, these errors lead to inflated genetic maps. In addition, map construction in many species is performed using full-sibling family populations derived from the outcrossing of two individuals, where unknown parental phase and varying segregation types further complicate construction. We present a new methodology for modeling low coverage sequencing data in the construction of genetic linkage maps using full-sibling populations of diploid species, implemented in a package called GUSMap. Our model is based on the Lander–Green hidden Markov model but extended to account for errors present in sequencing data. We were able to obtain accurate estimates of the recombination fractions and overall map distance using GUSMap, while most existing mapping packages produced inflated genetic maps in the presence of errors. Our results demonstrate the feasibility of using low coverage sequencing data to produce genetic maps without requiring extensive filtering of potentially erroneous genotypes, provided that the associated errors are correctly accounted for in the model.

## Abstract (precis)

Next generation sequencing-based genotyping platforms allow for the construction of high density genetic linkage maps. However, data generated using these platforms often contain errors resulting from miscalled bases and missing parental alleles that are due...

THE emergence of high-throughput sequencing methods that multiplex large numbers of individuals has provided a cost-effective approach to perform genome-wide genotyping and discovery of genetic variation. Two of the primary multiplexing sequencing methods are whole genome sequencing and reduced representation approaches, including whole-exome sequencing (Hodges *et al.* 2007), restriction-site associated DNA sequencing (Baird *et al.* 2008), and genotyping-by-sequencing (Elshire *et al.* 2011), among others (Heffelfinger *et al.* 2014). The introduction of these methods has led to the rapid increase in both the number of species being sequenced, especially nonmodel species (Ellegren 2014), and the number of markers available for analysis. Consequently, these methods provide opportunities to construct more dense genetic linkage maps compared with previous technologies, which is useful in scenarios where alternative high-density marker systems are infeasible (expensive to establish and validate). Genetic maps are important as they facilitate the investigation of many species in terms of their genes, such as associating phenotypes to the genome via QTL, validating assemblies, ordering contigs in assemblies, and performing comparative genome analyses (Cheema and Dicks 2009; Liu *et al.* 2014).

Constructing linkage maps using sequencing data is complicated by the presence of two types of missing data that can result when the sequencing depth is low. The first is a missing genotype resulting from no alleles being called, while the second consists of a heterozygous genotype being called as homozygous due to only one of the parental alleles being sequenced at a particular locus (Dodds *et al.* 2015; Fragoso *et al.* 2016). The latter type is particularly problematic as it usually behaves like a genotyping error, which increases the frequency of inferred recombinations and results in inflated linkage maps (Lincoln and Lander 1992; Cartwright *et al.* 2007; Cheema and Dicks 2009). Typically, genotyping errors resulting from low sequencing coverage are removed via filtering, such as setting genotypes with an associated read depth below some threshold value to missing (Gardner *et al.* 2014; Mousavi *et al.* 2016) or using genotype quality scores to discard uncertain genotype calls (Chen *et al.* 2014; Hyma *et al.* 2015; Mousavi *et al.* 2016). Nevertheless, this requires sequencing at a higher depth, which results in fewer individuals being sequenced and fewer utilized loci for a given cost, and can leave a large proportion of the original data unused. Several algorithms have been developed for imputing missing genotypes and correcting erroneous genotypes in low coverage genome-sequencing data (Spindel *et al.* 2013; Huang *et al.* 2014; Swarts *et al.* 2014; Fragoso *et al.* 2016); however, all of these algorithms are designed only for inbred populations and are not applicable to outcrossed full-sibling (full-sib) families. Recently, two software packages have been developed for performing linkage mapping in full-sib families using sequencing data. These are Lep-MAP (Rastas *et al.* 2013, 2016) and HighMap (Liu *et al.* 2014), both of which address the computational problem associated with high-density maps but are not specifically designed to handle low coverage sequencing data.

Another complication is the presence of sequencing errors, reads where the base has been called incorrectly, which also leads to inflated genetic distances if not taken into account. In contrast to errors caused by low read depth, sequencing errors can result in homozygotes being called as heterozygotes. One approach for removing sequencing errors involves detecting double recombinants at very short distances and either correcting the genotypes (*e.g.*, a double recombinant becomes nonrecombinant) or setting genotypes resulting in double recombinants as missing (van Os *et al.* 2005; Wu *et al.* 2008; Cheema and Dicks 2009; Liu *et al.* 2014). The problem with correcting double recombinants is the possibility of false positives, particularly if the chromosomal order is inaccurate (Wu *et al.* 2008), while erroneous genotype calls on the outside loci cannot be detected. An alternative is to account for these errors by including additional parameters in the model (Cartwright *et al.* 2007; Rastas *et al.* 2013), although estimation of these parameters is not always straightforward when the error rate is unknown.

Linkage mapping in plants has often been applied to inbred populations derived from the cross of two fully homozygous parents (*e.g.*, recombinant inbred lines, double haploids) (Grattapaglia and Sederoff 1994; Maliepaard *et al.* 1997), although this is dependent upon the breeding system of the species. For many plant species and most animal species, self-incompatibility, severe inbreeding depression, or long generation times prevent the production of inbred lines, where a commonly used alternative mapping population is an outbred full-sib family derived from the crossing of two unrelated individuals (Schneider 2005; Singh and Singh 2015). Examples where outbred populations have been particularly utilized in linkage mapping range from forest trees to forages (Devey *et al.* 1994; Grattapaglia and Sederoff 1994; Plomion *et al.* 1995; Wilcox *et al.* 2001; Butcher *et al.* 2002; Faville *et al.* 2004; Griffiths *et al.* 2013). However, building linkage maps in outcrossed populations is complicated by loci having different segregation types (STs; *e.g.*, the number of alleles segregating in each parent) and unknown parental phase (Maliepaard *et al.* 1997; Lu *et al.* 2004). An early approach for performing linkage mapping in these populations was the pseudotestcross strategy (Grattapaglia and Sederoff 1994), which maps the paternal and maternal meioses independently. An alternative approach, which uses all available information, is to model both meioses simultaneously using a multipoint likelihood model, provided there is a sufficient number of loci segregating in both parents (Van Ooijen 2011). One such model is the Lander–Green hidden Markov model (HMM) for general pedigrees (Lander and Green 1987). Although applicable to full-sib family populations, computation of the Lander–Green HMM is infeasible for moderate-to-large pedigrees (Thompson 2000). Several variants of this model derived specifically for full-sib family populations in diploid species have been suggested (Ling 2000; Wu *et al.* 2002; Tong *et al.* 2010), which reduces the computational complexity by exploiting the conditional independence between individuals given the parental phase.

In this article, we describe a new statistical method that adjusts for bias in map length estimation due to errors in genotypic data derived from sequencing. Our method is based on the Lander-Green HMM for full-sib families in diploid species (Ling 2000; Wu *et al.* 2002; Tong *et al.* 2010) that is applicable to multifamily and sex-specific situations, but includes an additional component to account for errors associated with sequencing data. The performance of the methodology presented here is tested and compared with existing full-sib family software packages, using simulations and a real sequencing data set.

## Materials and Methods

### ST and parental phase

In full-sib families, the combination of alleles found in the parental genotypes, referred to as ST, varies from locus to locus. A complete classification of all STs in a full-sib family for diploid species has been given by Maliepaard *et al.* (1997). For sequencing data in diploid species that typically consists only of SNPs, the number of different alleles found at a given locus in the parents is usually two. Consequently, the relevant STs are AB×AB, AB×AA, AB×BB, AA×AB, and BB×AB, where *A* denotes the reference allele, *B* denotes the alternate allele (maternal × paternal), and AA, AB, and BB denote the reference homozygous, heterozygous, and alternate homozygous genotypes, respectively. Using the standard nomenclature of Groover *et al.* (1994), we refer to the STs AB×AB as both-informative (BI), AB×AA and AB×BB as maternal-informative (MI), and AA×AB and BB×AB as paternal-informative (PI). To distinguish between the two MI and PI STs, we refer to AB×AA as MI*_A_*, AB×BB as MI*_B_*, AA×AB as PI*_A_*, and BB×AB as PI*_B_*. The STs of AA×AA, AA×BB, BB×AA, and BB×BB are also possible, although they are not usually classified as they provide no information of recombination in either parent, and are hence referred to as uninformative (U). Uninformative loci are included in this classification since it is possible for a locus to be uninformative in one family but informative in another.

We let $Z_{fjk1}$ denote the allele on the paternally derived chromosome and $Z_{fjk0}$ denote the allele on the maternally derived chromosome at locus *j* for parent *k* in family *f*, for f=1,…,F and j=1,…,M. *F* is the total number of families, *M* is the total number of loci, k=1 for the paternal parent, and k=0 for the maternal parent. The ordered parental genotype pair (OPGP) is defined as the unique combination of $Z_{fj11},$ $Z_{fj10},$ $Z_{fj01}$, and $Z_{fj00}.$ Across the four STs of BI, PI, MI, and U, there are 16 distinct OPGPs (Table 1). Specification of the OPGP for all loci is equivalent to determining the parental haplotypes and consequently the allelic phase of the parents.

**Table 1:** The OPGPs for both-informative, paternal-informative, maternal-informative, and uninformative biallelic loci in a full-sib family

ST	OPGP	$Z_{fj11}$	$Z_{fj10}$	$Z_{fj01}$	$Z_{fj00}$
BI	1	*A*	*B*	*A*	*B*
	2	*B*	*A*	*A*	*B*
	3	*A*	*B*	*B*	*A*
	4	*B*	*A*	*B*	*A*
PI*_A_*	5	*A*	*B*	*A*	*A*
	6	*B*	*A*	*A*	*A*
PI*_B_*	7	*A*	*B*	*B*	*B*
	8	*B*	*A*	*B*	*B*
MI*_A_*	9	*A*	*A*	*A*	*B*
	10	*A*	*A*	*B*	*A*
MI*_B_*	11	*B*	*B*	*A*	*B*
	12	*B*	*B*	*B*	*A*
U	13	*A*	*A*	*A*	*A*
	14	*A*	*A*	*B*	*B*
	15	*B*	*B*	*A*	*A*
	16	*B*	*B*	*B*	*B*

### Data and models

We begin by assuming that there are no errors present. In such a case, the data are denoted by $G_{fij},$ the true genotype call (AA, AB, or BB) for individual *i* in family *f* at locus *j*, where $i=1,\ldots ,N_{f}$ and $N_{f}$ is the number of individuals in family *f*. We denote the vector (length *N*) of true genotypes at locus *j* by $G_{\cdot \cdot j}={\left(G_{11j},\ldots ,G_{1N_{1}j},G_{21j},\ldots ,G_{FN_{F}j}\right)}^{T},$ where $N=\sum_{f=1}^{F}N_{f}$ and the superscript *T* denotes the transpose. The latent inheritance vectors are denoted $S_{fij}={\left(S_{fij1},S_{fij0}\right)}^{T},$ where $S_{fij1}$ is the inheritance from the paternal parent and $S_{fij0}$ is the inheritance from the maternal parent. The value of $S_{fijk}$ is 0 if the allele is derived from the parent’s maternal chromosome and 1 if the allele is derived from the parent’s paternal chromosome. We denote the inheritance vector (length 2N) for all individuals at locus *j* by $S_{\cdot \cdot j}={\left(S_{11j}^{T},\ldots ,S_{1N_{1}j}^{T},S_{21j}^{T},\ldots ,S_{FN_{F}j}^{T}\right)}^{T}.$

#### Lander–Green HMM:

For multilocus analysis in general pedigrees, Lander and Green (1987) proposed, using the HMM:

$$P\left(G\right)=\sum_{S}P\left(S_{\cdot \cdot 1}\right)P\left(G_{\cdot \cdot 1}|S_{\cdot \cdot 1}\right)\prod_{j=2}^{M}P\left(S_{\cdot \cdot j}|S_{\cdot \cdot j-1}\right)P\left(G_{\cdot \cdot j}|S_{\cdot \cdot j}\right),$$ (1)

where $S={\left(S_{\cdot \cdot 1}^{T},\ldots ,S_{\cdot \cdot M}^{T}\right)}^{T}.$ In HMM theory, $P\left(S_{\cdot \cdot j}|S_{\cdot \cdot j-1}\right)$ is known as the transmission probability, $P\left(G_{\cdot \cdot j}|S_{\cdot \cdot j}\right)$ as the emission probability, and $P\left(S_{\cdot \cdot 1}\right)$ as the initial distribution.

#### HMM for full-sib families:

In its original form, the Lander–Green HMM likelihood can be computed in $O\left(N^{2}M\right)$ steps using the forward-backward algorithm of Baum *et al.* (1970). Computing the Lander–Green HMM likelihood quickly becomes infeasible for pedigrees of moderate-to-large sizes. In full-sib family populations, individuals within and between families are conditionally independent given the OPGPs (*e.g.*, parental phases). The HMM for full-sib family populations is

$$P\left(G\right)=\prod_{f=1}^{F}\prod_{i=1}^{N_{f}}\left[\sum_{S_{fi\cdot }}P\left(S_{fi1}\right)P\left(G_{fi1}|S_{fi1},Z_{f1}\right)\prod_{j=2}^{M}P\left(S_{fij}|S_{fij-1}\right)P\left(G_{fij}|S_{fij},Z_{fj}\right)\right],$$ (2)

with $S_{fi\cdot }={\left(S_{fi1}^{T},\ldots ,S_{fiM}^{T}\right)}^{T}$ and $Z_{fj}={\left(Z_{fj11},Z_{fj10},Z_{fj01},Z_{fj00}\right)}^{T}.$ Using Equation 2, the computational time is reduced to O(NM), provided that the OPGPs are known for all families.

Let $r_{j1}$ and $r_{j0}$ denote the paternal and maternal recombination fraction, respectively, between locus *j* and locus j+1, where these recombination fractions are constrained to the interval [0,0.5]. For Equation 2, the transition probabilities are

$$P\left(S_{fij}|S_{fij-1}\right)=P\left(S_{fij1}|S_{fij-1\;1}\right)P\left(S_{fij0}|S_{fij-1\;0}\right),$$ (3)

where

$$P\left(S_{fijk}|S_{fij-1\;k}\right)=\{\begin{array}{ll} \left(1-r_{j-1\;k}\right), & S_{fijk}=S_{fij-1\;k},\\ r_{j-1\;k}, & S_{fijk}\neq S_{fij-1\;k}, \end{array}$$ (4)

and the emission probabilities are

$$P\left(G_{fij}=g|S_{fij}=\left(s_{1},s_{2}\right),Z_{fj}\right)=\{\begin{array}{ll} 1, & Z_{fj1s_{1}}⊕Z_{fj0s_{2}}=g,\\ 0, & \text{otherwise}, \end{array}$$ (5)

where ⊕ denotes the concatenation of two alleles to form a genotype such that A⊕B=B⊕A=AB. When the genotype is missing, the emission probability is $P\left(G_{fij}|S_{fij},Z_{fj}\right)=1$ for all inheritance vectors.

To compute the likelihood of the full-sib family HMM, forward recursion is used. Define $\alpha_{fij}\left(S_{fij}\right)$ as the forward probability which satisfies the relations

$$\alpha_{fi1}\left(S_{fi1}\right)=\pi_{fi}P\left(G_{fi1}|S_{fi1},Z_{f1}\right),$$ (6)

where $\pi_{fi}=P\left(S_{fi1}\right),$ and

$$\alpha_{fij}\left(S_{fij}\right)=\sum_{S_{fij-1}}\alpha_{fij-1}\left(S_{fij-1}\right)P\left(S_{fij}|S_{fij-1}\right)P\left(G_{fij}|S_{fij},Z_{fj}\right)$$ (7)

for j=2,…,M. Usually, the initial distribution is taken to be 2N independent Bernoulli trials (Ling 2000; Tong *et al.* 2010), so that $\pi_{fi}=1/4$ for all f,i. The likelihood of the HMM for individual *i* in family *f* is

$$L_{fi}=\sum_{S_{fiM}}\alpha_{fiM}\left(S_{fiM}\right).$$ (8)

As individuals within and between families are conditionally independent given the OPGPs of all the parents, the likelihood for multiple full-sib families is

$$L=\prod_{f=1}^{F}\prod_{i=1}^{N_{f}}L_{fi}.$$ (9)

In situations where some loci are uninformative in the maternal or paternal parent across all families, a slight adjustment to the parametrization of the model is required. If the paternal (maternal) genotype at locus j+1 is homozygous in every family or the paternal (maternal) genotypes at all loci from locus 1 to *j* are homozygous in every family, then the recombination fraction $r_{j1}$ ($r_{j0}$) cannot be estimated and is excluded from the model. Under this parametrization, the sex-specific recombination fraction $r_{j1}$ ($r_{j0}$) is now interpreted as the probability of a recombination in the paternal (maternal) parent between locus j+1 and the previous locus that is segregating in the paternal (maternal) parent. The model can also be specified with non sex-specific recombination fractions (*i.e.*, $r_{j1}=r_{j0}$), in which case this adjustment to the parametrization is not required.

#### Incorporating errors in the Lander–Green HMM:

When there is error present in the sequencing data, the genotypes$G_{fij}$ are latent. The observed data are the number of reads for the reference allele *A*, and alternate allele *B*. We denote the number of reads for the reference allele observed for individual *i* in family *f* at locus *j* by $Y_{fij},$ where $Y_{fij}$ is an integer value between 0 and $d_{fij},$ and $d_{fij}$ is the sequencing depth at locus *j* for individual *i* in family *f*. The sequencing depth, $d_{fij},$ is equal to the sum of the number of reads for the reference and alternate alleles. We denote the vector (length *N*) of reference allele counts at locus *j* by $Y_{\cdot \cdot j}={\left(Y_{11j},\ldots ,Y_{1N_{1}j},Y_{21j},\ldots ,Y_{FN_{F}j}\right)}^{T}.$ If $Y_{\cdot \cdot j}$ is conditionally independent between loci given $G_{\cdot \cdot j},$ which is a reasonable assumption if only a single locus on each read is chosen for linkage, then the extended HMM for sequencing data becomes

$$\begin{array}{c} P\left(Y\right)=\sum_{S}P\left(S_{\cdot \cdot 1}\right)\left(\sum_{G_{\cdot \cdot 1}}P\left(Y_{\cdot \cdot 1}|G_{\cdot \cdot 1}\right)P\left(G_{\cdot \cdot 1}|S_{\cdot \cdot 1}\right)\right)\\ \prod_{j=2}^{M}P\left(S_{\cdot \cdot j}|S_{\cdot \cdot j-1}\right)\left(\sum_{G_{\cdot \cdot j}}P\left(Y_{\cdot \cdot j}|G_{\cdot \cdot j}\right)P\left(G_{\cdot \cdot j}|S_{\cdot \cdot j}\right)\right). \end{array}$$ (10)

The transmission probabilities in Equation 10 are the same as in Equation 1. The emission probability, conditional on the sequencing depth $d_{fij},$ is $\sum_{G_{..j}}P\left(Y_{\cdot \cdot j}|G_{\cdot \cdot j}\right)P\left(G_{\cdot \cdot j}|S_{\cdot \cdot j}\right).$

#### Full-sib HMM for sequencing data:

If the number of reference alleles observed in the sequencing data, $Y_{fij},$ is conditionally independent between individuals given the true genotypes, $G_{fij},$ then the full-sib family HMM for sequencing data is

$$P\left(Y\right)=\prod_{f=1}^{F}\prod_{i=1}^{N_{f}}\left[\sum_{S_{fi\cdot }}P\left(S_{fi1}\right)\left(\sum_{G_{fi1}}P\left(Y_{fi1}|G_{fi1}\right)P\left(G_{fi1}|S_{fi1},Z_{f1}\right)\right)\prod_{j=2}^{M}P\left(S_{fij}|S_{fij-1}\right)\left(\sum_{G_{fij}}P\left(Y_{fij}|G_{fij}\right)P\left(G_{fij}|S_{fij},Z_{fj}\right)\right)\right].$$ (11)

The only change in Equation 11 compared with Equation 2 is the emission probabilities, which requires specifying the conditional probabilities $P\left(Y_{fij}|G_{fij}\right).$ Suppose that $Y_{fij}$ arises from a random binomial sample of the alleles found in $G_{fij}$ (Dodds *et al.* 2015) and suppose that sequencing errors occur independently between reads, then

$$\begin{array}{c} p_{AA}=P\left(Y_{fij}=a|G_{fij}=AA\right)=\left(\begin{array}{c} d_{fij}\\ a \end{array}\right){\left(1-\varepsilon \right)}^{a}\;\varepsilon^{d_{fij}-a}\\ p_{AB}=P\left(Y_{fij}=a|G_{fij}=AB\right)=\left(\begin{array}{c} d_{fij}\\ a \end{array}\right){\left(\frac{1}{2}\right)}^{d_{fij}}\\ p_{BB}=P\left(Y_{fij}=a|G_{fij}=BB\right)=\left(\begin{array}{c} d_{fij}\\ a \end{array}\right){\left(1-\varepsilon \right)}^{d_{fij}-a}\;\varepsilon^{a}. \end{array}$$ (12)

See Supplemental Material, File S1 for derivation of these probabilities. Under these assumptions, the emission probabilities for Equation 11 are

$$\sum_{G_{fij}}P\left(Y_{fij}|G_{fij}\right)P\left(G_{fij}|S_{fij}=\left(s_{1},s_{2}\right),Z_{fj}\right)=p_{Z_{fj1s_{1}}⊕Z_{fj0s_{2}}}.$$ (13)

Consequently, the likelihood of the HMM for sequencing data corresponds to Equation 9, with the emission probability $P\left(G_{fij}|S_{fij},Z_{fj}\right)$ replaced by $\sum_{G_{fij}}P\left(Y_{fij}|G_{fij}\right)P\left(G_{fij}|S_{fij},Z_{fj}\right).$

### Inferring OPGPs

The likelihoods for full-sib families derived in the previous sections assume that the OPGPs (or parental phases) are known. In practice, this information is unknown, although the OPGPs can, in some cases, be inferred from the grandparental genotype information. Nevertheless, if there is no grandparental information, then inference of the OPGPs using progeny genotypes (assuming parents are known and accurately genotyped) is required.

We initialize the value of $Z_{fj}$ for each locus to a default value, that is, we initialize $Z_{fj}={\left(A,B,A,B\right)}^{T}$ if the locus is BI, $Z_{fj}={\left(A,B,A,A\right)}^{T}$ if the locus is PI*_A_*, $Z_{fj}=c{\left(A,B,B,B\right)}^{T}$ if the locus is PI*_B_*, $Z_{fj}=c{\left(A,A,A,B\right)}^{T}$ if the locus is MI*_A_*, and $Z_{fj}=c{\left(B,B,A,B\right)}^{T}$ if the locus is MI*_B_*. Inference of the OPGPs for family *f* can be achieved by relaxing the constraint on $r_{j1}$ and $r_{j0}$ such that $r_{j1},r_{j0}\in \left[0,1\right]$ and maximizing the likelihood

$$L_{f}=\prod_{i=1}^{N_{f}}\sum_{S_{fiM}}\alpha_{fiM}\left(S_{fiM}\right),$$ (14)

where $\alpha_{fiM}\left(S_{fiM}\right)$ is defined as in Equations 6 and 7, and the emission probability is $P\left(G_{fij}|S_{fij},Z_{fj}\right)$ for inference under Equation 2, but $\sum_{G_{fij}}P\left(Y_{fij}|G_{fij}\right)P\left(G_{fij}|S_{fij},Z_{fj}\right)$ for inference under Equation 11. The OPGP of locus j=2,…,M can be inferred relative to the previous OPGPs based on whether the maximum likelihood estimates of $r_{j-1\;1}$ and/or $r_{j-1\;0}$ are greater or less than 0.5, where the OPGP for the first locus is set to a baseline value depending on its ST (see File S1 for details).

### Implementation

An implementation of the new methodology presented in this article can be found in the GUSMap (Genotyping Uncertainty with Sequencing data and linkage Mapping) software, which is freely available as a package for the programming language R (R Core Team 2017) and can be downloaded from https://github.com/tpbilton/GUSMap. Maximum likelihood estimates can be obtained using the expectation-maximization algorithm (Dempster *et al.* 1977) or directly, using numerical optimization (see File S1 for details). In this article, the expectation-maximization algorithm was used in all analyses.

### Software comparison

Using simulated and real data, the performance of GUSMap v0.1.1 (GM) was compared to the four linkage mapping software packages: CRI-MAP 2.507 (CM) (Green *et al.* 1990), JoinMap 4.1 (JM) (Van Ooijen 2011), Lep-MAP2 (Rastas *et al.* 2016), and OneMap v2.0-4 (OM) (Margarido *et al.* 2007), all of which are commonly used for full-sib family populations. In general, the default parameter settings were used, except for JM and Lep-MAP2. In JM, the threshold for determining linkage was set to zero in order for a complete map to be computed in every data set and the maximum likelihood algorithm was used. For Lep-MAP2, detection of duplicate loci was removed (argument removeDuplicates = 0). In addition, Lep-MAP2 includes estimation of an error parameter for each locus and was implemented using two sets of parameter options. The first corresponds to the model that includes error parameters, referred to as LM2*ε*, while the second corresponds to the exclusion of all error parameters (arguments learnErrorParameters = 0 and initError = 0) and is referred to as LM2. For all packages, non sex-specific recombination fractions ($r_{j1}=r_{j0}$) were computed.

With sequencing data, some genotype calls may result in apparent Mendelian errors, which occur when a genotype call for a PI*_A_* or MI*_A_* locus is homozygous for the alternate allele, or a genotype call for a PI*_B_* or MI*_B_* locus is homozygous for the reference allele. Genotype calls determined to be a Mendelian error were set as heterozygous, since the packages CM, JM, and OM either cannot handle data sets with these errors present or output warning messages. Mendelian errors were not corrected with GM as they are accounted for in the HMM. In addition, some heterozygous genotype calls in the sequencing data were supported by over nine reads for one allele but only a single read for the other allele. As these genotype calls are likely to be sequencing errors, they were set to missing for the standard packages, but not for GM, as they provide information used to estimate the sequencing error parameter *ε*.

### Simulation

Sequencing data were simulated using the following procedure. Inheritance vectors for progeny were generated based on the true parental recombination values, assuming no interference and equal probability of the first locus being derived from either parent. These inheritance vectors were converted to genotype calls for a prespecified set of OPGPs. From these genotype calls, simulated sequencing data sets were generated as follows: a sequencing depth at each locus in each individual was generated by simulating realizations from a negative binomial distribution with mean $\mu_{d_{j}}$ and dispersion parameter of 2:

$$P\left(d_{fij}=d\right)=\frac{\Gamma \left(d+\delta \right)}{d!\Gamma \left(\delta \right)}{\left(\frac{\delta }{\mu_{d_{j}}+\delta }\right)}^{\delta }{\left(\frac{\mu_{d_{j}}}{\mu_{d_{j}}+\delta }\right)}^{d},$$ (15)

where δ=2, $\mu_{d_{j}}$ is the mean sequencing depth for locus *j*, and Γ(⋅) denotes the gamma function. A sample of $d_{fij}$ alleles are found by randomly sampling the alleles of the true genotype, $G_{fij},$ with replacement, where a miscall of the sampled allele (*e.g.*, a *B* allele called as *A* and vice versa) occurred with probability *ε*.

Two sets of simulations were conducted. In the first set, the performance of the five software packages is examined and compared under different mean read depths and sequencing error rates. This set consisted of simulating a 1000 single full-sib families (F=1) with 100 offspring ($N_{1}=100$), 12 loci (M=12), and a fixed recombination rate of 1% in both parents ($r_{j1}=r_{j0}=0.01$). The STs and OPGPs of the loci are given in Table S1 in File S2. Different combinations of mean read depth $\mu_{d_{j}},$ and sequencing error *ε* were used, where the mean read depth was either low ($\mu_{d_{j}}=2$), moderate ($\mu_{d_{j}}=10$), or high ($\mu_{d_{j}}=20$), and the sequencing error rate was either absent (ε=0), small (ε=0.002), or large (ε=0.01). To remove errors associated with low sequencing depth, the simulated data were filtered such that all genotype calls with an associated read depth below some threshold were set to missing. The threshold used was 11 for the high depth setting ($\mu_{d_{j}}=20$), six for the moderate depth setting ($\mu_{d_{j}}=10$), and was not applied for the low depth setting ($\mu_{d_{j}}=2$) since an insufficient number of nonmissing genotypes would remain. This filtering step was not performed for GM as it models undercalled heterozygous genotypes.

The second set of simulations investigates the optimal sequencing depth for a given sequencing effort (defined as the number of individuals times the number of loci times the mean read depth). The parameters used in this set corresponded to those in the previous set, with the exception that the sequencing error rate was fixed at 0.2% (ε=0.002), the number of individuals was varied, and the mean read depth was set such that an average sequencing effort of 10,000 was maintained. Recombination fractions were estimated using GM, assuming a known OPGP.

The code for implementing the simulations is found in File S3. GM, CM, LM2, LM2*ε*, and OM were all run on a Linux desktop computer with four Intel Core i7-870 central processing units running at 2.93 GHz frequency, while JM was run on a Windows 10 Enterprise desktop computer with four Intel Core i7-3770 central processing units running at 3.40 GHz frequency. As JM has no scripting functionality, it was automated using a custom C# script coupled with a coded user interface test, a program which can automate mouse clicks and keyboard strokes on Windows operating systems.

### Mānuka data

A single full-sib biparental family (n=180) of mānuka (*Leptospermum scoparium*, J.R. Forst. and G. Forst.; Myrtaceae) derived from a reciprocal pair-cross of heterozygous individuals, was genotyped along with the parents, using a genotyping-by-sequencing approach (Elshire *et al.* 2011). Samples consisted of young expanding leaves collected from 3-month-old seedlings grown in the glasshouse. Two genotyping-by-sequencing libraries were prepared based on the Elshire method (Elshire *et al.* 2011), using a double digest with the restriction enzymes *Ape*KI/*Msp*I and sequenced at AgResearch, Invermay, Animal Genomics laboratory. A size selection step was performed on the DNA such that the genomic part of each read was between 27 and 377 bp. The samples were sequenced on an Illumina HiSeq 2500 v4 chemistry producing 1 × 100 single end reads. Each genotyping-by-sequencing library was sequenced on two lanes of a flow cell generating ∼29.2 Gbp of raw sequence data per lane. The parents were run on both lanes to obtain higher sequencing depths, while each progeny was run on one of the two lanes. Quality control was performed using DECONVQC (https://github.com/AgResearch/DECONVQC) and KGD (Dodds *et al.* 2015). Three progeny samples were discarded, one due to having a sample call rate <0.05 and two others due to being identified as duplicate samples. Sequence reads were mapped using Bowtie2 version 2.1.0 (Langmead and Salzberg 2012) and SNP variants were called using Tassel3 version 3.0.173 (Bradbury *et al.* 2007).

To compare GM to the other packages, only variants called on chromosome 11 were retained for further analysis, with additional filtering performed as follows. SNPs with a minor allele frequency <0.05 or 20% or more missing genotypes (*e.g.*, a read depth of zero) were discarded (6205 in total). The ST of each SNP was inferred based on the parental genotypes provided that the read depth for both parents was >5, where 603 SNPs were discarded because the ST could not be inferred. A segregation test was performed on each SNP using the chi-square test, where a *P*-value of 0.05 was used and the expected counts were adjusted for low read depths (see File S1 for details). This resulted in another 964 SNPs being discarded. To ensure that each read only contained a single variant, adjacent SNPs were placed into bins if the distance separating them was <180 bp, with one SNP from each bin retained for the final analysis by random selection. A further 401 SNPs were removed leaving 680 SNPs with 270 PI, 294 MI, and 116 BI loci. These data are available in the R package GUSMap. The percentiles of the distribution of the mean depth across SNPs and individuals are given in Table S2 in File S2. The level of filtering used here is typical of sequencing data (with the exception of no depth filtering) and was chosen to aid comparison between GUSMap and the other four packages.

To assess SNP ordering on chromosome 11, heatmaps of two-point recombination fraction estimates between all SNPs segregating in the same parent were produced. GM was used to compute the two-point estimates (with ε=0), where the phase between the SNP pair was taken as the one which maximized the likelihood value. Linkage maps were computed using two independent sets of SNPs: a low depth set consisting of all the SNPs with a mean read depth below six and a high depth set which was obtained by setting all genotype calls with a read depth below 20 to missing and selecting all the SNPs, such that a call rate of at least 80% was maintained. In total, there were 95 low depth SNPs and 54 high depth SNPs.

### Data availability

The mānuka data set used in this article is available in the software package GUSMap (https://github.com/tpbilton/GUSMap). Code for generating the simulated data are found in File S3. Supplementary methods are given in File S1 and supplementary tables and figures are given in File S2.

## Results

### Simulations

**Figure 1:**
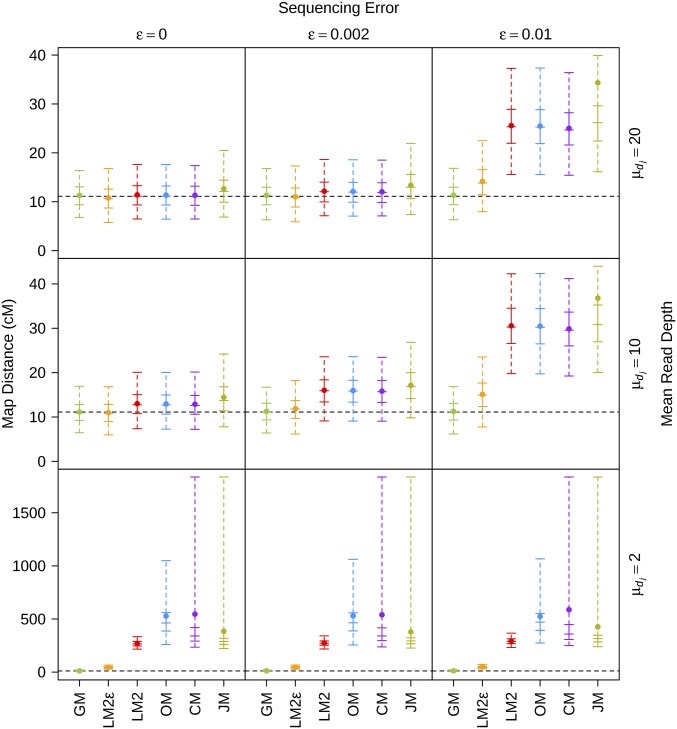
Distribution of the map distance estimates for the first set of simulations across varying mean read depths (rows) and varying sequencing error rates (columns). The solid point represents the mean; the vertical solid line represents the interquartile range; the vertical dashed line represents the range between the 2.5th and 97.5th percentiles; the five horizontal solid lines represent, in ascending order, the 2.5th percentile, lower quantile, median, upper quantile, and 97.5th percentile; and the horizontal black dotted line represents the true parameter value. Map distances are in centimorgans and were computed using the Haldane mapping function.

The distribution of the overall map distance estimates obtained using the various software packages in the first set of simulations is given in Figure 1, while the distribution of the recombination fraction estimates for each simulation are given in Figures S1–S9 in File S2. Across all simulations, LM2, OM, and CM performed similarly and at high depth with no sequencing error, gave relatively unbiased estimates. However, at moderate depth with no sequencing error, the overall map distance estimates from LM2, OM, and CM were slightly larger than the true value, which suggests that the cut-off of six has not removed all errors associated with low sequencing depth. The bias in the map distance and recombination fraction estimates increased as the level of sequencing error increased for the high and moderate depth scenarios. In comparison, JM produced maps that were on average slightly longer and more biased across all the low and moderate depth simulations. These inflated maps seem to be driven by biases in the recombination fraction estimates for $r_{4}$ and $r_{5}$ (see Figures S1–S6 in File S2), particularly when the sequencing error was small or absent. These parameters all include one locus in the region between locus 4 and locus 6, where a PI locus is between two MI loci. As JM uses only a three-point approach, the lack of informativeness between adjacent loci in this region may explain the observed bias.

LM2*ε* was able to produce accurate estimates of the recombination fractions and overall map distance when the sequencing error was absent or low for the moderate and high read depth scenarios. Nevertheless, when the sequencing error was large, LM2*ε* produced biased estimates of the overall map distance, which was driven by large biases in the recombination fraction estimates that include an outside locus (see Figures S3 and S6 in File S2). At low depth, the four existing software packages all gave very map distance estimates across all of the various sequencing error rates, which was expected given the large number of errors in the data. Of these methods, LM2*ε* performed the best although its map distance estimates were still ∼4–5 times larger than the true value (Figure S10 in File S2). In addition, the recombination fraction estimates for LM2*ε* at low depth were biased (see Figures S7–S9 in File S2), although for the middle sections of the map the bias was in both directions resulting in less inflation of the overall map distance but a distortion of the SNP distribution across the linkage map. In contrast, GM was the only package which was able to give accurate estimates of the overall map distance and recombination fractions across all simulation scenarios.

**Figure 2:**
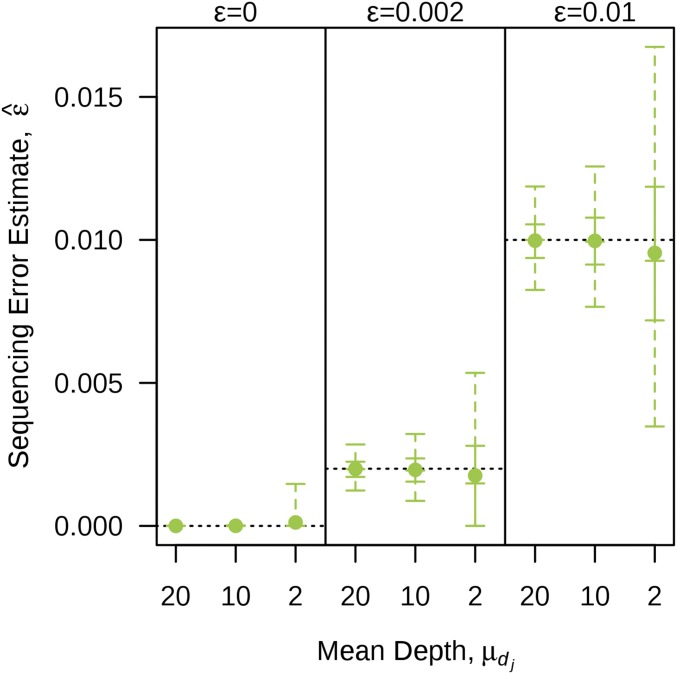
Distribution of sequencing error estimates obtained from GM for various combinations of mean read depths and sequencing error rates. The solid point represents the mean; the vertical solid line represents the interquartile range; the vertical dashed line represents the range between the 2.5th and 97.5th percentiles; the five horizontal solid lines represent, in ascending order, the 2.5th percentile, lower quantile, median, upper quantile, and 97.5th percentile; and the horizontal black dotted lines represent the true parameter values.

The distribution of the sequencing error estimates obtained from GM are given in Figure 2. For the high and moderate depth simulations, the estimates were relatively accurate, while there was a small bias for the low depth simulations. The variability in the estimates increased as the mean read depth decreased, which was expected given there was more variability in the data at low depths.

**Figure 3:**
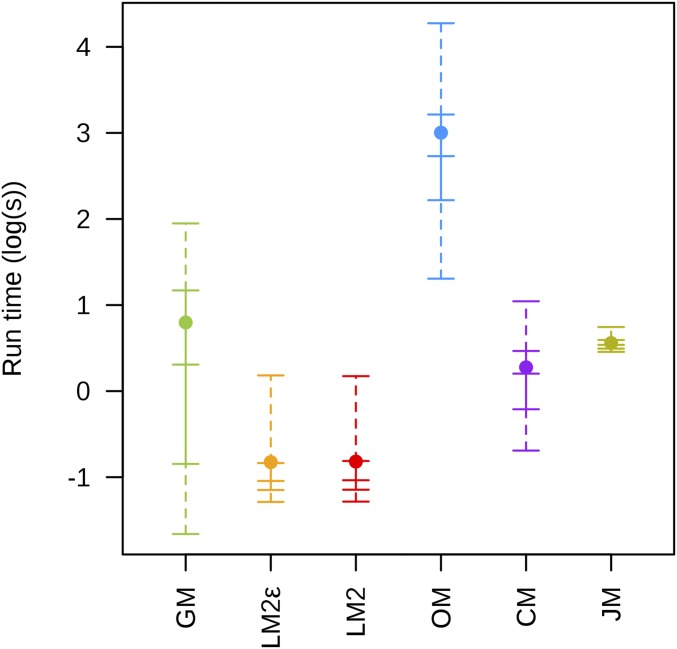
Distribution of log transformed computational time (in seconds) used on each data set across all nine simulation scenarios for the first set of simulations and each software package. The solid point represents the mean; the vertical solid line represents the interquartile range; the vertical dashed line represents the range between the 2.5th and 97.5th percentiles; and the five horizontal solid lines represent, in ascending order, the 2.5th percentile, lower quantile, median, upper quantile, and 97.5th percentile.

Figure 3 gives the distribution of computation time required for each package across all scenarios of the first set of simulations. Of all the packages, LM2 was the fastest, regardless of whether the error parameters were included, while CM, GM, and OM were ∼3, ∼5, and ∼45 times slower than LM2, respectively. As JM is a nonscripting program, providing a sensible measure of computation time is difficult. For these simulations, the time recorded was only for the step to compute the map, which on average required four times more time than LM2, but did not include the extensive user interaction time needed to import the data and create the required nodes.

The percentage of data sets in which the vector of OPGPs was correctly inferred in the first set of simulations is displayed in Table 2. For the moderate and high depth simulations, all packages apart from JM were able to correctly infer the OPGPs, regardless of the amount of sequencing error present. For the low depth simulations, only GM and LM2*ε* were able to correctly infer phase across all the simulations, while OM rarely inferred phase correctly and LM2 incorrectly inferred phase for a few data sets. There were a small number of phasing errors for JM across the various scenarios, with the frequency of these errors increasing as the number of erroneous genotypes increased. For CM, phase inference was not required since it implements the Lander–Green HMM for general pedigrees.

**Table 2:** Percentage of simulated data sets in which the vector of OPGPs was correctly inferred

Mean depth	Sequencing error	Software package				
$\mu_{d_{j}}$	*ε*	GM	LM2*ε*	LM2	OM	JM
20	0	100.0	100.0	100.0	100.0	99.9
	0.002	100.0	100.0	100.0	100.0	100.0
	0.01	100.0	100.0	100.0	100.0	98.8
10	0	100.0	100.0	100.0	100.0	99.9
	0.002	100.0	100.0	100.0	100.0	100.0
	0.01	100.0	100.0	100.0	100.0	99.4
2	0	100.0	100.0	99.9	2.8	94.0
	0.002	100.0	100.0	99.6	3.6	94.8
	0.01	100.0	100.0	99.6	4.5	93.2

**Figure 4:**
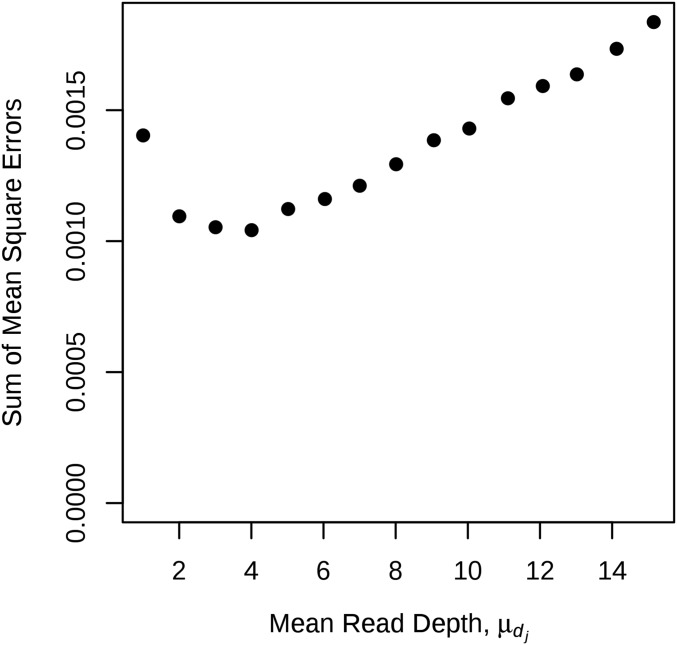
Sum of recombination fraction estimates mean square errors for fixed sequencing effort. Recombination fraction estimates were computed using GM, where the OPGPs was known and the sequencing effort was fixed at 10,000 reads. The parameters used to generate the data sets corresponds to the first set of simulations, with the exception that the mean depth and number of individuals were set to maintain a sequencing effort of 10,000 reads. The sum of the mean square errors was calculated using $\sum_{j=1}^{11}MSE\left({\hat{r}}_{j}\right).$ The number of individuals range from 833 for a mean depth of 1 to 55, for a mean depth of 15.15.

For the second simulation set, the sum of the mean square errors of the recombination fraction estimates verses the mean depth is given in Figure 4. This plot suggests that the optimal sequencing depth was around three or four as the smallest mean square error occurred around these depths.

### Mānuka data

Heatmaps of two-point recombination fraction estimates for SNPs located on chromosome 11 are given in Figure S11A (paternally segregating SNPs) and Figure S11B (maternally segregating SNPs) in File S2. A number of SNPs appeared either to be incorrectly ordered on the chromosome or located on the wrong chromosome and were discarded from the analysis (164 in total). Heatmaps of the remaining SNPs (Figure S11, C and D in File S2) suggest that the order of these SNPs was fairly accurate. For this analysis, we assume that this order is correct.

**Figure 5:**
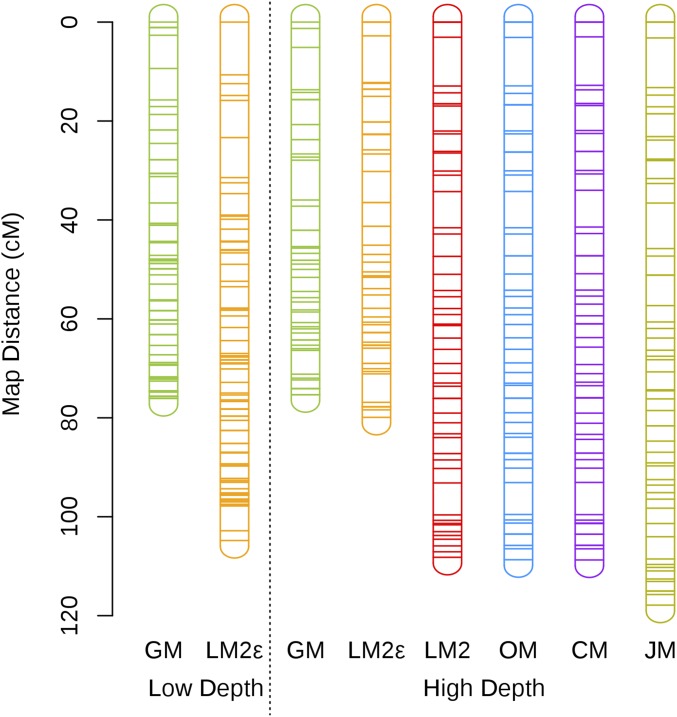
Subset of linkage maps for SNPs on chromosome 11 of mānuka computed using the various software packages. Low depth refers to the maps produced using SNPs with a mean read depth below 6, while high depth refers to maps produced using SNPs with <20% missing data after setting genotypes with a read depth below 20 to missing. Map distances are in centimorgans and were computed using the Haldane mapping function. The rounded rectangles represent the chromosomes and the horizontal lines represent the SNPs. Different sets of SNPs are used in the low and high depth sets. See Figure S13 in File S2 for a plot of the genetic distance verses the physical distance for each these maps.

Linkage maps of chromosome 11 were computed for both the low depth and high depth set of SNPs using GM and the standard software packages. These linkage maps are given in Figure S12 in File S2 (all maps) and Figure 5 (maps that were <150 cM), with the overall map distance estimates given in Table 3. For the low depth set, the maps obtained from LM2, OM, CM, and JM were between eight and nine times longer compared to the high depth set. These inflated map estimates were expected given the substantial proportion of undercalled heterozygous genotypes present at low depth and is consistent with the simulation results. Compared to GM and LM2*ε* at high depth, LM2*ε* produced a map that was ∼20 cM longer when using the low depth SNPs, with large distances between SNPs at the chromosome ends. For the high depth setting, the maps produced by LM2*ε* and GM were similar in length and shorter than the maps obtain using LM2, OM, CM, and JM by ∼30 cM. This suggests that there was sequencing error present in this data set, where both LM2*ε* and GM are accounting for these errors. The overall map distance estimated using GM was consistent across both SNP sets at ∼76 cM, with estimated sequencing error rates of 0.32% for the low depth SNPs and 0.20% for the high depth SNPs. Overall, these results resemble those observed in the simulations and suggests that GM has accounted for most of the errors present in both the low and high depth settings. In terms of phasing, all packages inferred the same phase under both SNP sets, apart from CM which does not require parental phase to compute the recombination fractions.

**Table 3:** Overall map distance estimates (centimorgan) for chromosome 11 of mānuka

SNP set	GM	LM2*ε*	LM2	OM	CM	JM
Low depth	76.1	104.8	990.0	989.1	977.1	950.7
High depth	75.3	79.9	108.2	108.7	108.7	117.9

Map distances were computed using the Haldane mapping function.

## Discussion

We have developed a new statistical method for constructing genetic maps from a set of ordered loci on outcrossed full-sib families in diploid species that have been genotyped using multiplexing sequencing methods. Our methodology uses a HMM approach to overcome the issues associated with mapping in full-sib families and to account for errors resulting from low sequencing depth and miscalled bases. In addition, our methodology is applicable to multi-family and sex-specific situations and has been implemented in the software package GUSMap.

Simulation results show that GUSMap was able to accurately estimate the recombination fractions and overall map distance for varying sequencing error rates and mean read depth scenarios. In contrast, most of the other software packages were unable to produce reasonable results when there were errors present in the data, resulting in biased estimates. Specifically, the overall map distances obtained were inflated, which is known to occur in linkage mapping when genotypes errors are present and not taken into account (Lincoln and Lander 1992; Cartwright *et al.* 2007). Of all the standard software programs, the implementation of Lep-MAP2 that included error parameters was least sensitive to map inflation and able to provide reasonable results when the number of erroneous genotypes was not too large. However, in low coverage settings, it still contained substantial bias in the map distance estimates and distortion in the SNP distribution across the map. Maps produced by CM, OM, and Lep-MAP2 without an error parameter were similar since these packages are essentially implementing the same model. In comparison, JM tended to produce maps that were slightly longer in length.

The mānuka analysis suggests that GUSMap performs well under real-life low depth sequencing scenarios. This observation is based on the fact that GUSMap produced consistent estimates of the overall map distance from two independent set of SNPs with different sequencing coverage, and gave a similar map length compared with LM2*ε* at high depth. In contrast, the other packages all produced hugely inflated genetic maps, except for LM2*ε*, although there still appeared to be some inflation under the low depth setting. This analysis shows that GUSMap is able to reduce map inflation caused by errors in the data and provide better linkage maps and estimates of overall map distance, particularly in low coverage scenarios.

Of the software packages considered in this paper, only Lep-MAP2 and GUSMap account for errors using information provided by the observed data. For Lep-MAP2, estimation of these errors seems to be based on detecting double recombinants. Thus, the error parameters for the end loci are always zero, since double recombinants cannot be counted, resulting in bias for recombination fraction estimates that include an outside locus when these loci contain erroneous genotype calls. Furthermore, for situations when many errors are present in the data, such as low coverage data, determining which genotypes are incorrect based on double recombinants is difficult. GUSMap, on the other hand, uses the allele count information to model errors due to missing parental alleles and sequencing errors. In particular, it is able to differentiate between the two error types, allowing it to produce accurate maps in low coverage scenarios. What is more, GUSMap can account for errors associated with low read depths in a two-point analysis, which is not the case when error estimation is based on detecting double recombinants. This is useful for producing heatmaps of two-point recombination fraction estimates to examine chromosomal ordering. GUSMap also uses only a single parameter to model sequencing error, whereas LM2*ε* specifies a separate error parameter for each locus. Consequently, GUSMap makes the assumption that the sequencing error rate is constant across individuals and loci. In practice, this assumption may not hold, although the mānuka results suggest that it may be reasonable in some situations.

Simulation results suggest that GUSMap is able to provide reasonably accurate estimates of the sequencing error rate, although there was a small bias at low depth. For the mānuka data, the estimates from GUSMap suggest that between 0.2 and 0.3% of the reads in the filtered data were sequencing errors, although there was discrepancy in the sequencing error rates estimated between the two SNP sets. This discrepancy could be due to natural variation since the sequencing error estimates at low depth are highly variable. Alternatively, the high depth SNPs with large sequencing errors may have been removed through the filtering process as detection of these SNPs would be easier since the sequencing errors are not confounded with errors resulting from low depth.

Nearly all full-sib family software packages require inferring parental phase. Phasing errors can result in estimates that are close to or equal to 0.5. GUSMap and Lep-MAP2 were mostly able to correctly infer phase across all the simulations. Both of these packages use a similar phasing approach in that they infer phase based on sex-specific recombination fractions estimated on the interval [0,1] using a multipoint likelihood containing all the loci. In contrast, OM failed to correctly infer phase in the low depth simulations, which suggests that phase inference based on maximizing the likelihood value is unreliable in the presence of severe model misspecification. JM also failed to infer phase in some data sets, which suggests that phasing using a multipoint approach can be superior to using a three-point approach. The ability to correctly infer phase also depends on a number of factors; namely, the density of the markers, the family size, and for sequencing data, the average sequencing depth. Simulation results (Figure S14 in File S2) suggest that GUSMap is able to infer phase for low coverage data provided the maps are at moderate-to-high density, the mean read depth is at least two, and there are at least 25 progeny in the family.

A number of assumptions have been made in the methodology we have outlined. First, the order of the loci is assumed to be known beforehand, which is often not the case with sequencing data, particularly for *de novo* assemblies. One approach to ordering loci is to evaluate the likelihood under different chromosomal orders, where the best order is the one that gives the highest likelihood value. This approach is feasible for improving order locally, provided that the initial order is fairly accurate, but is impractical for ordering large number of loci that are randomly ordered. A reasonable initial order could be computed by combining two-point estimates obtained from GUSMap with existing ordering algorithms. More research is required to investigate loci ordering in low coverage settings. Another assumption is that all the parental genotypes are known for all loci, so that the STs can be determined unequivocally. In practice, all the parents will be sequenced using multiplexing methods and therefore are subject to the same type of genotyping errors as the progeny. One way to circumvent this issue is to sequence the parents multiple times to obtain higher sequencing depths, although this still results in some loci having insufficient depths to accurately infer the ST. Alternatively, if there is a sufficient number of individuals in each family, the ST of each locus could be inferred from progeny genotypes using a segregation test. Other assumptions include independence of the reads observed in the sequencing data between loci, which is a reasonable assumption provided there is only a single locus on each read, and the sampling of the alleles from the true genotype is random. For the latter assumption, the probabilities in Equation 12 can be adjusted to reflect any prior knowledge of the sampling of the true genotypes (*e.g.*, preferential sampling of alleles). This methodology is limited to autosomal biallelic loci in diploid species or functionally diploid species (*e.g.*, allopolyploids). Extension of this methodology to allosomal (sex-linked) loci and multiallelic loci (*e.g.*, microsatellite markers) would require deriving the correct emission probabilities in Equation 12 for the HMM.

GUSMap provides researchers with a tool to compute genetic maps from a set of ordered loci using sequencing data and overcomes a number of issues related to this data. First, it is able to handle varying sequencing depths across SNPs, which is typical of sequencing data, allowing more SNPs to be utilized that would otherwise be discarded in a high depth analysis. Second, SNPs called in the bioinformatics process must meet a minimal set of filtering criteria, which is aimed at removing erroneous genotypes and fictitious SNPs. GUSMap removes the need for filtering genotyping calls based on read depth information or skewed apparent segregation and correcting erroneous genotypes through detecting double recombinants. This allows researchers to use low coverage data, especially when cost constraints may prohibit the production of sufficiently high coverage data, to construct genetic maps. Another advantage of GUSMap is its use of a statistical approach to model errors, which allows it to be combined with existing statistical techniques to make inference on model parameters, such as quantifying the rate of sequencing errors, and assessing modeling assumptions. Although the methodology of GUSMap is derived specifically for outbred full-sib populations, it can also be applied to inbred backcross populations, since the ST of all the loci are either PI or MI, and inbred F2 populations where the ST of all the loci are BI.

## Supplementary Material

Supplemental material is available online at www.genetics.org/lookup/suppl/doi:10.1534/genetics.117.300627/-/DC1.
