# Supplementary material for: Accounting for Errors in Low Coverage High-Throughput Sequencing Data When Constructing Genetic Maps Using Biparental Outcrossed Populations
Source: Genetics. 2018 Feb 27;209(1):65–76. doi: 10.1534/genetics.117.300627 (PMC5937187; doi:10.1534/genetics.117.300627)
Supplement: Supplementary file 1 [file 65FileS1.pdf]

Supplementary Methods: Accounting for Errors in Low Coverage High-Throughput Sequencing Data when Constructing Genetic Maps using Biparental Outcrossed Populations

Timothy P. Bilton[*,†], Matthew R. Schofield[*], Michael A. Black[‡], David Chagné[‡,§], Phillip L. Wilcox[*] and Ken G. Dodds[†]

[*]Department of Mathematics and Statistics, University of Otago, Dunedin, New Zealand
[†]Invermay Agricultural Centre, AgResearch, Mosgiel, New Zealand
[‡] Department of Biochemistry, University of Otago, Dunedin, New Zealand
[§] Palmerston North Research Centre, New Zealand Institute for Plant & Food Research Limited (Plant & Food Research), Palmerston North, New Zealand

Contents

1 Derivation of emission probabilities for sequencing data **2**

2 Algorithm for inferring OPGPs **3**

3 GUSMap Optimization Procedures **3**

3.1 EM approach 3

3.1.1 Forward and backward probabilities 5

3.1.2 Complete data likelihood 6

3.1.3 E-Step 7

3.1.4 M-Step 7

3.2 BFGS approach 8

1 Derivation of emission probabilities for sequencing data

In this section, we derive the conditional probabilities $P(Y_{fij}|G_{fij})$. For a biallelic loci, there are four possible ways in which a given read my occur which are,

1. Paternally derived allele is sequenced without error.
2. Paternally derived allele is sequenced with error.
3. Maternally derived allele is sequenced without error
4. Maternally derived allele is sequenced with error

These four cases are shown in Figure A1.

For a given read, we denote the probability that the cth case has occurred by q_c and we denote the probability that the reference allele is sequenced by p_A. If the true genotype is homozygous for the reference allele ($X^p = X^m = A$), then

$$p_A = q_1 + q_3 = \frac{1}{2}(1-\varepsilon) + \frac{1}{2}(1-\varepsilon) = (1-\varepsilon).$$

If the true genotype is AB, then

$$p_A = q_1 + q_4 = \frac{1}{2}(1-\varepsilon) + \frac{1}{2}\varepsilon = \frac{1}{2},$$

if the reference allele is paternally derived ($X^p = A$, $X^m = B$) and

$$p_A = q_2 + q_3 = \frac{1}{2}\varepsilon + \frac{1}{2}(1-\varepsilon) = \frac{1}{2}$$

if the reference allele is maternally derived ($X^p = B$, $X^m = A$). If the true genotype is homozygous for the alternate allele ($X^p = X^m = B$), then

$$p_A = q_2 + q_4 = \frac{1}{2}\varepsilon + \frac{1}{2}\varepsilon = \varepsilon.$$

The probability of observing a reference alleles for individual i in family f at locus j given the true genotype, G_{fij}, follows a binomial distribution where there are d_{fij} trials and the

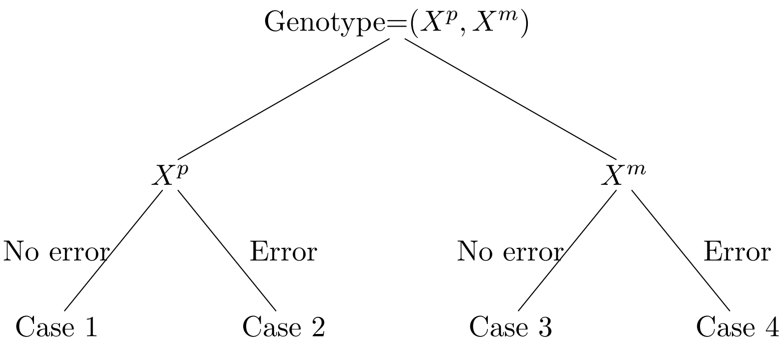

Figure A1: Four possible ways an allele may arise in a biallelic loci for a full-sib family. X^p denotes the paternally derived allele and X^m denotes the maternally derived allele.

probability of successfully sampling the reference allele is p_A, that is,

$$P(Y_{fij} = a | G_{fij}) = \binom{d_{fij}}{a} p_A^a (1 - p_A)^{d_{fij} - a},$$

which results in the probabilities given in Eq (12).

2 Algorithm for inferring OPGPs

The OPGP of locus j, for $j = 2, \ldots, M$, can be inferred relative to the previous OPGPs using Algorithm 1, where $I(\cdot)$ denotes the indicator function.

3 GUSMap Optimization Procedures

Two optimization procedures have been implemented in GUSMap, the Expectation-Maximization (EM) approach and the 'BFGS' method as implemented in the R function **optim()**.

3.1 EM approach

In the derivations that follow in this section, we will denote the emission probabilities using $P(O_{fij}|\boldsymbol{S}_{fij}, \boldsymbol{Z}_{fj})$, where O_{fij} denotes the observed data at locus j for individual i in family f. The emission probability $P(O_{fij}|\boldsymbol{S}_{fij}, \boldsymbol{Z}_{fj})$ is equal to $P(G_{fij}|\boldsymbol{S}_{fij}, \boldsymbol{Z}_{fj})$ for HMM (2) and $\sum_{G_{fij}} P(Y_{fij}|G_{fij}) P(G_{fij}|\boldsymbol{S}_{fij}, \boldsymbol{Z}_{fj})$ for the HMM for sequencing data defined in model (11).

Algorithm 1: Reconstructing OPGPs from sex-specific recombination fraction estimates in family f

Input : r_{j1} and r_{j0} for $j = 1, \ldots, M-1$

Initialize: Set

$$\boldsymbol{Z}_{f1} = \begin{cases} (A,B,A,B)^T & \text{if locus 1 is BI} \\ (A,B,A,A)^T & \text{if locus 1 is PI}_A \\ (A,B,B,B)^T & \text{if locus 1 is PI}_B \\ (A,A,A,B)^T & \text{if locus 1 is MI}_A \\ (B,B,A,B)^T & \text{if locus 1 is MI}_B \end{cases}$$

Iterate : **for** $j = 2, \ldots, M$ **do**
- Set $c = \max(k)$ such that $Z_{fk11} \neq Z_{fk10}$ for $k = 1, \ldots, j-1$
- Set $d = \max(k)$ such that $Z_{fk01} \neq Z_{fk00}$ for $k = 1, \ldots, j-1$
- **if** locus j is PI **then**
  - **if** locus j is PI_A **then**
    - Set $Z_{fj01} = A$ and $Z_{fj00} = A$
  - **else**
    - Set $Z_{fj01} = B$ and $Z_{fj00} = B$
  - **if** all loci from 1 to $j-1$ are MI **then**
    - Set $Z_{fj11} = A$ and $Z_{fj10} = B$
  - **else if** $r_{j-11} > 0.5$ **then**
    - Set $Z_{fj11} = Z_{fc10}$ and $Z_{fj10} = Z_{fc11}$
  - **else**
    - Set $Z_{fj11} = Z_{fc11}$ and $Z_{fj10} = Z_{fc10}$
- **else if** locus j is MI **then**
  - **if** locus j is MI_A **then**
    - Set $Z_{fj11} = A$ and $Z_{fj10} = A$
  - **else**
    - Set $Z_{fj11} = B$ and $Z_{fj10} = B$
  - **if** all loci from 1 to $j-1$ are PI **then**
    - Set $Z_{fj01} = A$ and $Z_{fj00} = B$
  - **else if** $r_{j-10} > 0.5$ **then**
    - Set $Z_{fj01} = Z_{fd00}$ and $Z_{fj00} = Z_{fd01}$
  - **else**
    - Set $Z_{fj01} = Z_{fd01}$ and $Z_{fj00} = Z_{fd00}$
- **else if** locus j is BI **then**
  - **if** $r_{j-11} > 0.5$ **then**
    - Set $Z_{fj11} = Z_{fc10}$ and $Z_{fj10} = Z_{fc11}$
  - **else**
    - Set $Z_{fj11} = Z_{fc11}$ and $Z_{fj10} = Z_{fc10}$
  - **else if** $r_{j-10} > 0.5$ **then**
    - Set $Z_{fj01} = Z_{fd00}$ and $Z_{fj00} = Z_{fd01}$
  - **else**
    - Set $Z_{fj01} = Z_{fd01}$ and $Z_{fj00} = Z_{fd00}$

Return : $\boldsymbol{Z}_{fj}$ for $j = 1, \ldots, M$

3.1.1 Forward and backward probabilities

The forward probabilities required for the EM algorithm are defined in Eqs (6) and (7), while the backward probabilities are defined recursively as,

$$\beta_{fiM}(\boldsymbol{S}_{fiM}) = 1, \tag{A1}$$

and,

$$\beta_{fij}(\boldsymbol{S}_{fij}) = \sum_{\boldsymbol{S}_{fij+1}} P(\boldsymbol{S}_{fij+1}|\boldsymbol{S}_{fij})P(O_{fij+1}|\boldsymbol{S}_{fij+1}, \boldsymbol{Z}_{fj+1})\beta_{fij+1}(\boldsymbol{S}_{fij+1}), \tag{A2}$$

for $j = 1, \ldots, M-1$. In HMM, computation of the forward and backward probabilities typically suffers from underflow issues when M gets sufficiently large. One way to overcome this issue is to scale the forward and backward probabilities as follows. Specifically, we define the scaled forward probabilities as

$$\hat{\alpha}_{fi1}(\boldsymbol{S}_{fi1}) = c_{fi1}\alpha_{fi1}(\boldsymbol{S}_{fi1}), \tag{A3}$$

and

$$\hat{\alpha}_{fij}(\boldsymbol{S}_{fij}) = c_{fij} \sum_{\boldsymbol{S}_{fij-1}} \hat{\alpha}_{fij-1}(\boldsymbol{S}_{fij-1})P(\boldsymbol{S}_{fij}|\boldsymbol{S}_{fij-1})P(O_{fij}|\boldsymbol{S}_{fij}, \boldsymbol{Z}_{fj}), \tag{A4}$$

for $j = 2, \ldots, M$, where the scaling coefficients are

$$c_{fi1} = \frac{1}{\sum_{\boldsymbol{S}_{fi1}} \alpha_{fi1}(\boldsymbol{S}_{fi1})}, \tag{A5}$$

and

$$c_{fij} = \frac{1}{\sum_{\boldsymbol{S}_{fij}} \sum_{\boldsymbol{S}_{fij-1}} \hat{\alpha}_{fij-1}(\boldsymbol{S}_{fij-1})P(\boldsymbol{S}_{fij}|\boldsymbol{S}_{fij-1})P(O_{fij}|\boldsymbol{S}_{fij}, \boldsymbol{Z}_{fj})}, \tag{A6}$$

for $j = 2, \ldots, M$. Similarly, the scaled backward probabilities are

$$\hat{\beta}_{fiM}(\boldsymbol{S}_{fiM}) = c_{fiM}, \tag{A7}$$

and

$$\hat{\beta}_{fij}(\boldsymbol{S}_{fij}) = c_{fij} \sum_{\boldsymbol{S}_{fij+1}} P(\boldsymbol{S}_{fij+1}|\boldsymbol{S}_{fij})P(O_{fij+1}|\boldsymbol{S}_{fij+1}, \boldsymbol{Z}_{fj+1})\hat{\beta}_{fij+1}(\boldsymbol{S}_{fij+1}), \tag{A8}$$

for $j = 1, \ldots, M-1$. Under this scaling scheme, we have that

$$\hat{\alpha}_{fij}(\boldsymbol{S}_{fij}) = \left[\prod_{t=1}^{j} c_{fit}\right] \alpha_{fij}(\boldsymbol{S}_{fij}), \tag{A9}$$

and

$$\hat{\beta}_{fij}(\boldsymbol{S}_{fij}) = \left[\prod_{t=j}^{M} c_{fit}\right] \beta_{fij}(\boldsymbol{S}_{fij}). \tag{A10}$$

The likelihood can then be computed using the scaling coefficients via

$$L_{fi} = \prod_{j=1}^{M} c_{fij}. \tag{A11}$$

3.1.2 Complete data likelihood

For the EM algorithm, the complete log likelihood can be derived as follows. Let $\boldsymbol{H}_{fij}$ represent the true hidden inheritance vector at locus j for individual i in family f. Define,

$$u_{fij}(\boldsymbol{S}_{fij}) = \begin{cases} 1 & \boldsymbol{S}_{fij} = \boldsymbol{H}_{fij} \\ 0 & \text{otherwise.} \end{cases} \tag{A12}$$

and

$$v_{fij}(\boldsymbol{S}_{fij}, \boldsymbol{S}_{fij+1}) = \begin{cases} 1 & \boldsymbol{S}_{fij} = \boldsymbol{H}_{fij} \text{ and } \boldsymbol{S}_{fij+1} = \boldsymbol{H}_{fij+1} \\ 0 & \text{otherwise.} \end{cases} \tag{A13}$$

Then the complete likelihood for the HMM is,

$$P(\boldsymbol{O}, \boldsymbol{H}|\boldsymbol{\theta}) = \prod_{f=1}^{F}\prod_{i=1}^{N_f} \pi_{fij} \prod_{j=1}^{M-1} P(\boldsymbol{S}_{fij+1}|\boldsymbol{S}_{fij})^{v_{fij}(\boldsymbol{S}_{fij}, \boldsymbol{S}_{fij+1})} \prod_{j=1}^{M} P(O_{fij}|\boldsymbol{S}_{fij})^{u_{fij}(\boldsymbol{S}_{fij})} \tag{A14}$$

where $\boldsymbol{O} = (O_{111}, \ldots, O_{11M}, O_{211}, \ldots, O_{FN_fM})^T$, $\boldsymbol{H} = (\boldsymbol{H}_{111}, \ldots, \boldsymbol{H}_{11M}, \boldsymbol{H}_{211}, \ldots, \boldsymbol{H}_{FN_fM})^T$ and $\boldsymbol{\theta} = (r_{11}, r_{10}, \ldots, r_{M-11}, r_{M-10}, \varepsilon)^T$.

3.1.3 E-Step

The expectation step requires computing the the conditional probabilities of observing the hidden variables $\boldsymbol{S}_{fij}$ given the observed data and the given parameter values. Specifically,

$$\begin{aligned}\hat{u}_{fij}(\boldsymbol{S}_{fij}) = P(\boldsymbol{S}_{fij}|\boldsymbol{O},\boldsymbol{\theta}) &= \frac{\alpha_{fij}(\boldsymbol{S}_{fij})\beta_{fij}(\boldsymbol{S}_{fij})}{L_{fi}} \\ &= \frac{\hat{\alpha}_{fij}(\boldsymbol{S}_{fij})\hat{\beta}_{fij}(\boldsymbol{S}_{fij})}{c_{fij}},\end{aligned} \tag{A15}$$

for $j = 1, \ldots, M$ and

$$\begin{aligned}\hat{v}_{fij}(\boldsymbol{S}_{fij}, \boldsymbol{S}_{fij+1}) &= P(\boldsymbol{S}_{fij}, \boldsymbol{S}_{fij+1}|\boldsymbol{O},\boldsymbol{\theta}) \\ &= \frac{\alpha_{fij}(\boldsymbol{S}_{fij})P(\boldsymbol{S}_{fij+1}|\boldsymbol{S}_{fij})P(O_{fij+1}|\boldsymbol{S}_{fij+1}, \boldsymbol{Z}_{fj+1})\beta_{fij+1}(\boldsymbol{S}_{fij+1})}{L_{fi}} \\ &= \hat{\alpha}_{fij}(\boldsymbol{S}_{fij})P(\boldsymbol{S}_{fij+1}|\boldsymbol{S}_{fij})P(O_{fij+1}|\boldsymbol{S}_{fij+1}, \boldsymbol{Z}_{fj+1})\hat{\beta}_{fij+1}(\boldsymbol{S}_{fij+1}).\end{aligned} \tag{A16}$$

for $j = 1, \ldots, M - 1$.

3.1.4 M-Step

Replacing the hidden variables $u_{fij}(\boldsymbol{S}_{fij})$ and $v_{fij}(\boldsymbol{S}_{fij}, \boldsymbol{S}_{fij+1})$ with $\hat{u}_{fij}(\boldsymbol{S}_{fij})$ and $\hat{v}_{fij}(\boldsymbol{S}_{fij}, \boldsymbol{S}_{fij+1})$ and maximizing the complete data likelihood (A14) with respect to the parameters yields the following expressions for maximizing the parameters given the complete data:

- The recombination fractions:

$$\hat{r}_{jk} = \frac{1}{2N}\sum_{f=1}^{F}\sum_{i=1}^{N_f}\hat{v}_{fij}(\boldsymbol{S}_{fij}, \boldsymbol{S}_{fij+1})n_k(\boldsymbol{S}_{fij}, \boldsymbol{S}_{fij+1}), \tag{A17}$$

  where

$$n_k(\boldsymbol{S}_{fij}, \boldsymbol{S}_{fij+1}) = \begin{cases}1 & S_{fijk} \neq S_{fij+1k} \\ 0 & S_{fijk} = S_{fij+1k}.\end{cases} \tag{A18}$$

- Sequencing error:

$$\hat{\varepsilon} = \frac{P}{P+Q} \tag{A19}$$

where

$$P = \sum_{f=1}^{F} \sum_{i=1}^{N_f} \sum_{j=1}^{M} u_{fij}(\boldsymbol{S}_{fij}) \left[aI(G_{fij} = BB) + (d-a)I(G_{fij} = AA) \right], \tag{A20}$$

and

$$Q = \sum_{f=1}^{F} \sum_{i=1}^{N_f} \sum_{j=1}^{M} u_{fij}(\boldsymbol{S}_{fij}) \left[aI(G_{fij} = AA) + (d-a)I(G_{fij} = BB) \right], \tag{A21}$$

where $I(\cdot)$ is the indicator function.

3.2 BFGS approach

The 'BFGS' method, as implemented in the **optim()** function, is an unconstrained numeric optimizer. Thus, since the recombination fraction and sequencing error parameters are only valid on a constrained region, the optimization needs to be performed on transformed parameter values which do not have any constraints. The transformations which achieve this are,

$$\rho(r) = \ln\left(\frac{2r}{1-2r}\right)$$

for the recombination fractions in all the likelihoods except for likelihood (14) where the logit transformation is used. The logit transformation is also used for the sequencing error parameter, ε. The maximum likelihood estimates for the parameters are computed by back transforming the transformed parameter estimates. To overcome underflow issues, the scaled forward probabilities as given in Equations (A3) and (A4) are used in the computation of the likelihood, while the likelihood functions are written in C to reduce computational time.

4 Segregation test for low depth data

Let G^*_{fij} denote the genotype observed in the sequencing data for individual i in family f at locus j. Assuming that G^*_{fij} arises from a random binomial sample of the alleles found in G_{fij}, then

$$\begin{aligned}
P(G^*_{fij} = AA|G_{fij} = AA) &= 1 \\
P(G^*_{fij} = AA|G_{fij} = AB) &= K_{fij} \\
P(G^*_{fij} = AB|G_{fij} = AB) &= 1 - 2K_{fij} \\
P(G^*_{fij} = BB|G_{fij} = AB) &= K_{fij} \\
P(G^*_{fij} = BB|G_{fij} = BB) &= 1,
\end{aligned} \tag{A22}$$

where $K_{fij} = 1/2^{d_{fij}}$ [1]. The probability of observing a major homozygous genotype at locus j for individual i in family f in the sequencing data can be expressed as

$$\begin{aligned}
P(G^*_{fij} = AA) &= \sum_{G_{fij}} P(G^*_{fij}|G_{fij})P(G_{fij}) \\
&= P(G^*_{fij} = AA|G_{fij} = AA)P(G_{fij} = AA) \\
&\quad + P(G^*_{fij} = AA|G_{fij} = AB)P(G_{fij} = AB) \\
&= P(G_{fij} = AA) + K_{fij}P(G_{fij} = AB),
\end{aligned}$$

In like manner, the probability of observing a minor homozygous genotype at locus j for individual i in family f can be expressed as

$$\begin{aligned}
P(G^*_{fij} = BB) &= P(G^*_{fij} = BB|G_{fij} = BB)P(G_{fij} = BB) \\
&\quad + P(G^*_{fij} = BB|G_{fij} = AB)P(G_{fij} = AB) \\
&= P(G_{fij} = BB) + K_{fij}P(G_{fij} = AB),
\end{aligned}$$

while the probability of observing a heterozygous genotype at locus j for individual i in family f can be expressed as

$$\begin{aligned}
P(G^*_{fij} = AB) &= P(G^*_{fij} = AB|G_{fij} = AB)P(G_{fij} = AB) \\
&= (1 - 2K_{fij})\, P(G_{fij} = AB).
\end{aligned}$$

From the above equations, we have that

$$P(G^*_{fij} = AA) = \begin{cases} \dfrac{1}{2} + K_{fij} & \text{if the locus is PI}_A \text{ or MI}_A \\ \dfrac{1}{4} + K_{fij} & \text{if the locus is BI} \\ K_{fij} & \text{if the locus is PI}_B \text{ or MI}_B \end{cases}$$

$$P(G^*_{fij} = AB) = \frac{1}{2} - 2K_{fij}$$

$$P(G^*_{fij} = BB) = \begin{cases} K_{fij} & \text{if the locus is PI}_A \text{ or MI}_A \\ \dfrac{1}{4} + K_{fij} & \text{if the locus is BI} \\ \dfrac{1}{2} + K_{fij} & \text{if the locus is PI}_B \text{ or MI}_B \end{cases}$$

Let M_f denote the number of individuals in family f which have a non-missing genotype. The expected counts of the genotype g at locus j in family f for sequencing data is given by $e_{fj}(g) = \sum_{i=1}^{M_f} P(G^*_{fij} = g)$. Furthermore, denote the observed counts for genotype g at locus j in family f for sequencing data by $o_{fj}(g)$. The chi-square statistic for the segregation test at locus j for family f with low depth data is

$$X^2 = \sum_{g \in (AA,AB,BB)} \frac{(o_{fj}(g) - e_{fj}(g))^2}{e_{fj}(g)}$$

which follows a chi-square distribution with 2 degrees of freedom. Thus, locus j is in segregation distortion (and therefore discarded) if the chi-square statistic is larger than the quantile of the chi-square distribution with 2 degrees of freedom corresponding to a specified significance level.

References

[1] Dodds KG, McEwan JC, Brauning R, Anderson RM, Van Stijn TC, Kristjánsson T, et al. Construction of relatedness matrices using genotyping-by-sequencing data. BMC Genomics. 2015;16:1047.
