# Supplementary material for: Accounting for Errors in Low Coverage High-Throughput Sequencing Data When Constructing Genetic Maps Using Biparental Outcrossed Populations
Source: Genetics. 2018 Feb 27;209(1):65–76. doi: 10.1534/genetics.117.300627 (PMC5937187; doi:10.1534/genetics.117.300627)
Supplement: Supplementary file 2 [file 65FileS2.pdf]

Table S1: Segregation type (ST) and ordered parental genotype pair (OPGP) for loci used in the first set of simulations

	Locus											
	**1**	**2**	**3**	**4**	**5**	**6**	**7**	**8**	**9**	**10**	**11**	**12**
ST	PI	BI	BI	MI	PI	MI	BI	BI	MI	BI	PI	MI
OPGP	5	1	2	9	6	10	3	4	9	1	6	1

See Table 1 for definition of the parental-informative (PI), maternal-informative (MI) and both-informative (BI) segregation types.

Table S2: Sample quantiles of the distribution for the mean read depth of the individuals and SNPs in the mānuka chromosome 11 data set

	Quantiles				
	**2.5%**	**25%**	**50%**	**75%**	**97.5%**
Individuals	4.42	12.36	20.13	26.62	46.01
SNPs	2.90	7.36	16.62	31.40	53.97

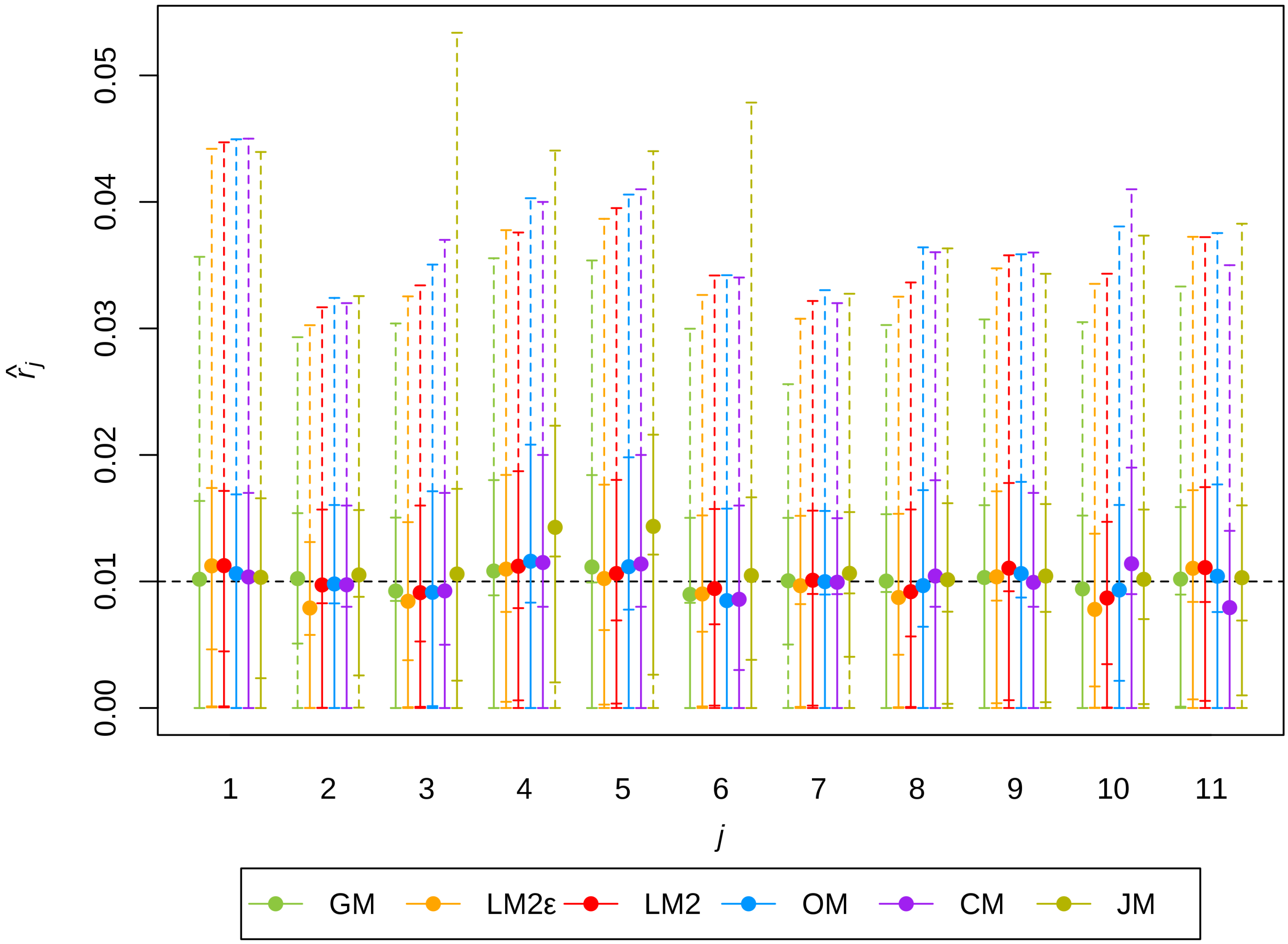

Figure S1: Distribution of the recombination fraction estimates, $\hat{r}_j$, for high depth and no sequencing error. The solid point represents the mean, the vertical solid line represents the interquartile range, the vertical dashed line represents the range between the 2.5^{th} and 97.5^{th} percentiles, the five horizontal solid lines represent, in ascending order, the 2.5^{th} percentile, lower quantile, median, upper quantile and 97.5^{th} percentile, and the horizontal black dotted line represents the true parameter value. j is the interval from the jth locus to the $(j+1)^{\text{th}}$ locus. The mean read depth was $\mu_{d_j} = 20$ and the sequencing error rate was $\varepsilon = 0$.

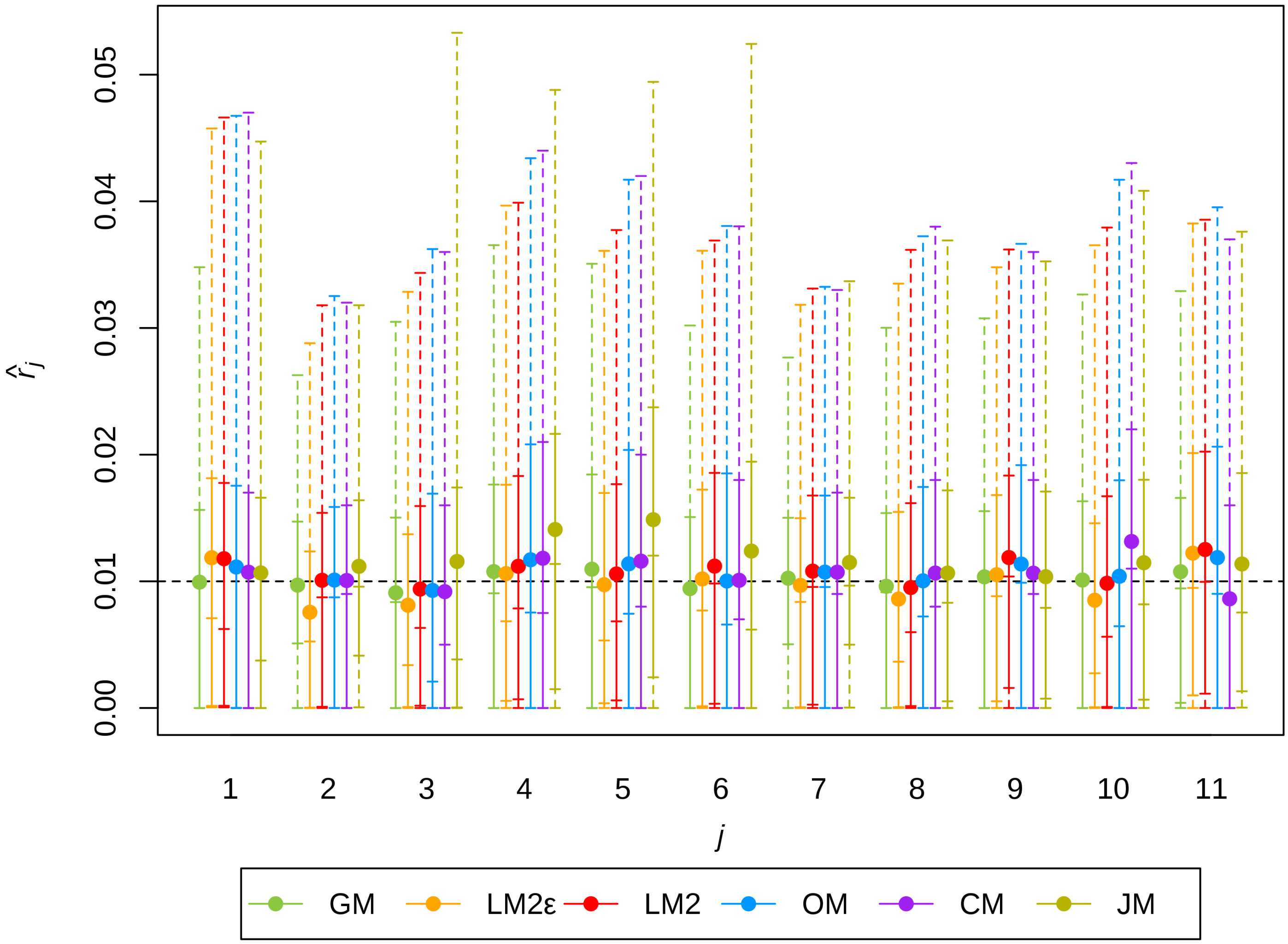

Figure S2: Distribution of the recombination fraction estimates, $\hat{r}_j$, for high depth and small sequencing error. The solid point represents the mean, the vertical solid line represents the interquartile range, the vertical dashed line represents the range between the 2.5^{th} and 97.5^{th} percentiles, the five horizontal solid lines represent, in ascending order, the 2.5^{th} percentile, lower quantile, median, upper quantile and 97.5^{th} percentile, and the horizontal black dotted line represents the true parameter value. j is the interval from the jth locus to the $(j+1)^{\text{th}}$ locus. The mean read depth was $\mu_{d_j} = 20$ and the sequencing error rate was $\varepsilon = 0.002$.

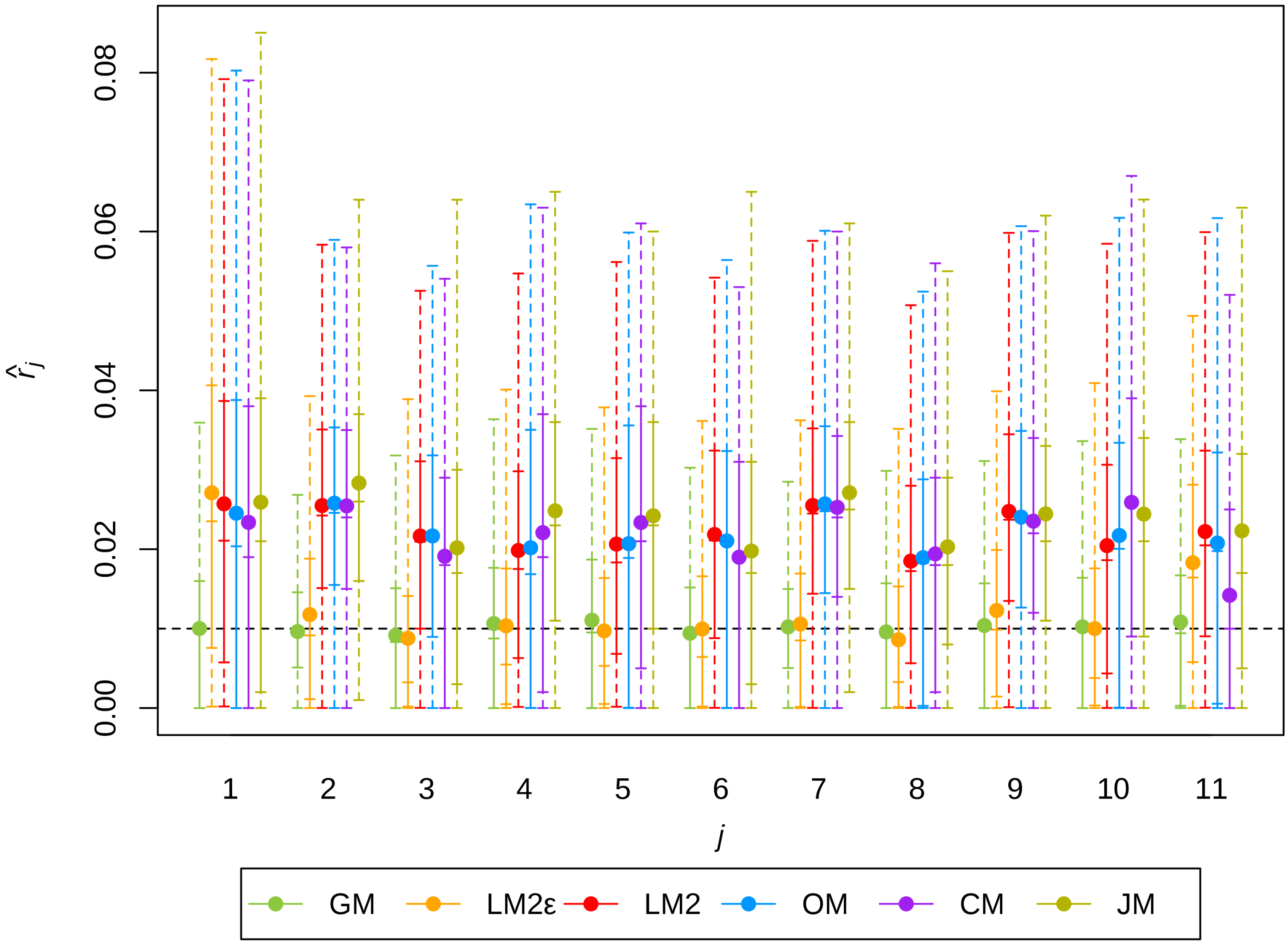

Figure S3: Distribution of the recombination fraction estimates, $\hat{r}_j$, for high depth and large sequencing error. The solid point represents the mean, the vertical solid line represents the interquartile range, the vertical dashed line represents the range between the 2.5^{th} and 97.5^{th} percentiles, the five horizontal solid lines represent, in ascending order, the 2.5^{th} percentile, lower quantile, median, upper quantile and 97.5^{th} percentile, and the horizontal black dotted line represents the true parameter value. j is the interval from the jth locus to the $(j+1)^{\text{th}}$ locus. The mean read depth was $\mu_{d_j} = 20$ and the sequencing error rate was $\varepsilon = 0.01$.

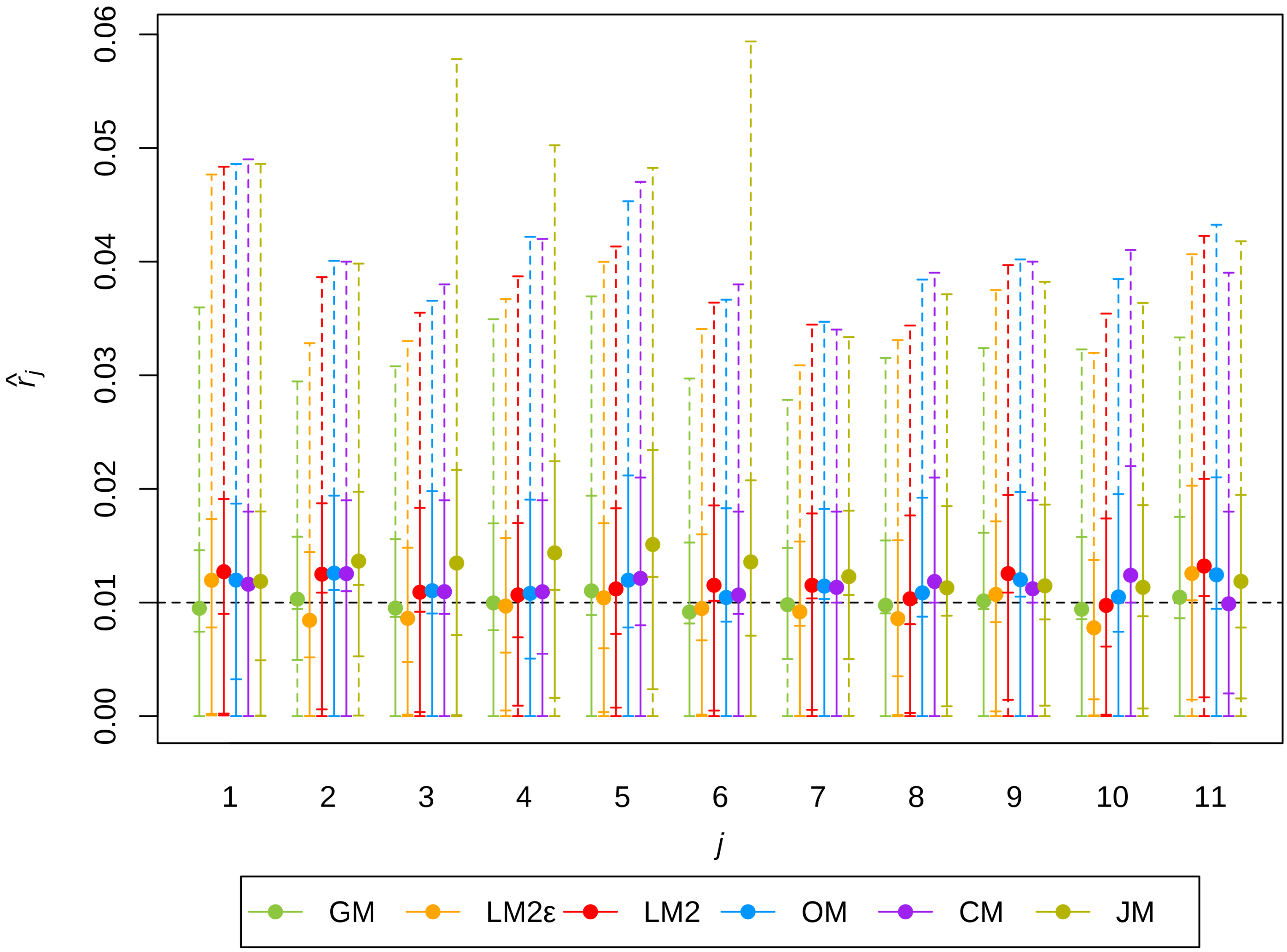

Figure S4: Distribution of the recombination fraction estimates, $\hat{r}_j$, for moderate depth and no sequencing error. The solid point represents the mean, the vertical solid line represents the interquartile range, the vertical dashed line represents the range between the 2.5^{th} and 97.5^{th} percentiles, the five horizontal solid lines represent, in ascending order, the 2.5^{th} percentile, lower quantile, median, upper quantile and 97.5^{th} percentile, and the horizontal black dotted line represents the true parameter value. j is the interval from the jth locus to the $(j+1)^{\text{th}}$ locus. The mean read depth was $\mu_{d_j} = 10$ and the sequencing error rate was $\varepsilon = 0$.

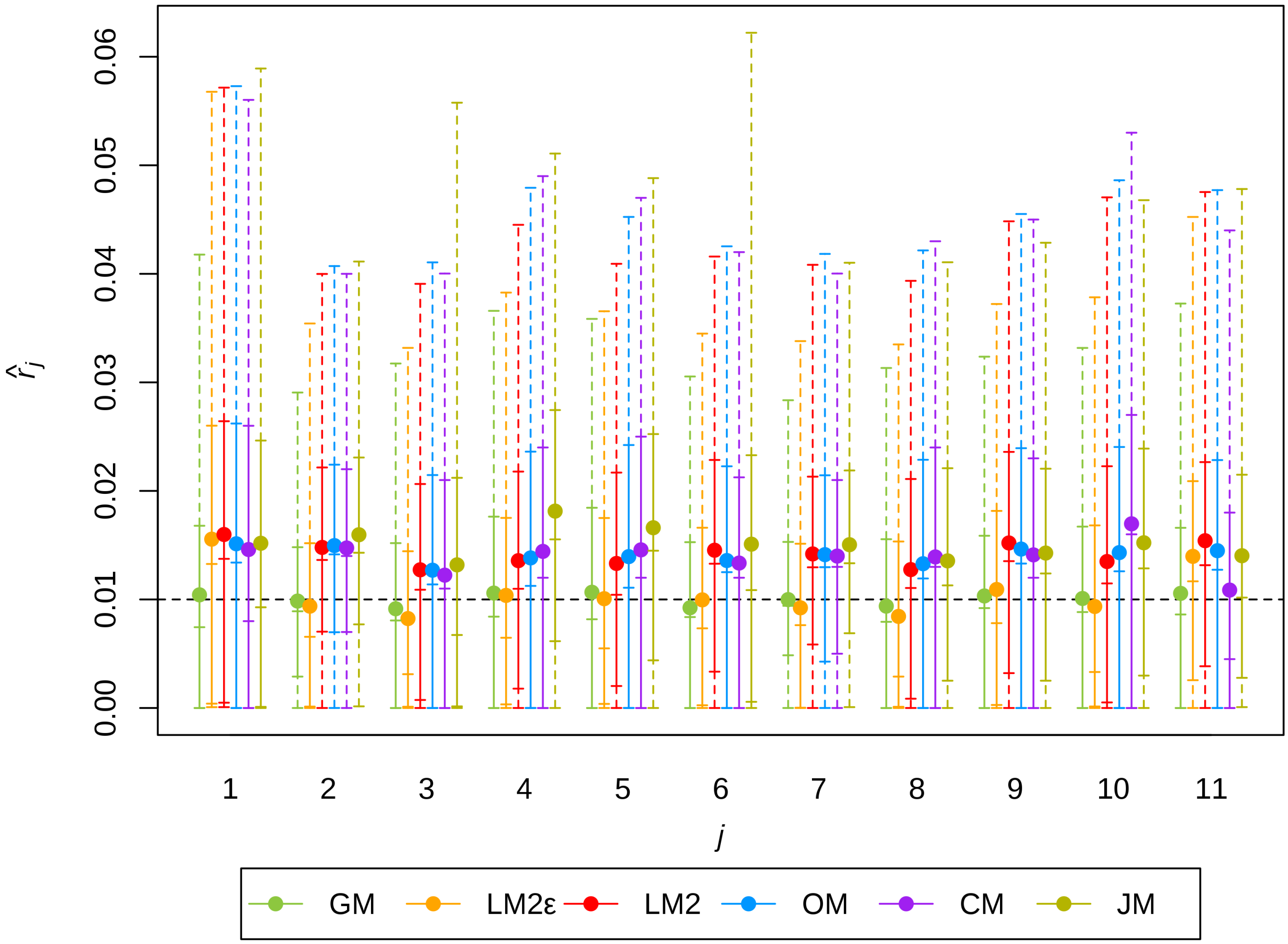

Figure S5: Distribution of the recombination fraction estimates, $\hat{r}_j$, for moderate depth and small sequencing error. The solid point represents the mean, the vertical solid line represents the interquartile range, the vertical dashed line represents the range between the 2.5^{th} and 97.5^{th} percentiles, the five horizontal solid lines represent, in ascending order, the 2.5^{th} percentile, lower quantile, median, upper quantile and 97.5^{th} percentile, and the horizontal black dotted line represents the true parameter value. j is the interval from the jth locus to the $(j+1)^{\text{th}}$ locus. The mean read depth was $\mu_{d_j} = 10$ and the sequencing error rate was $\varepsilon = 0.002$.

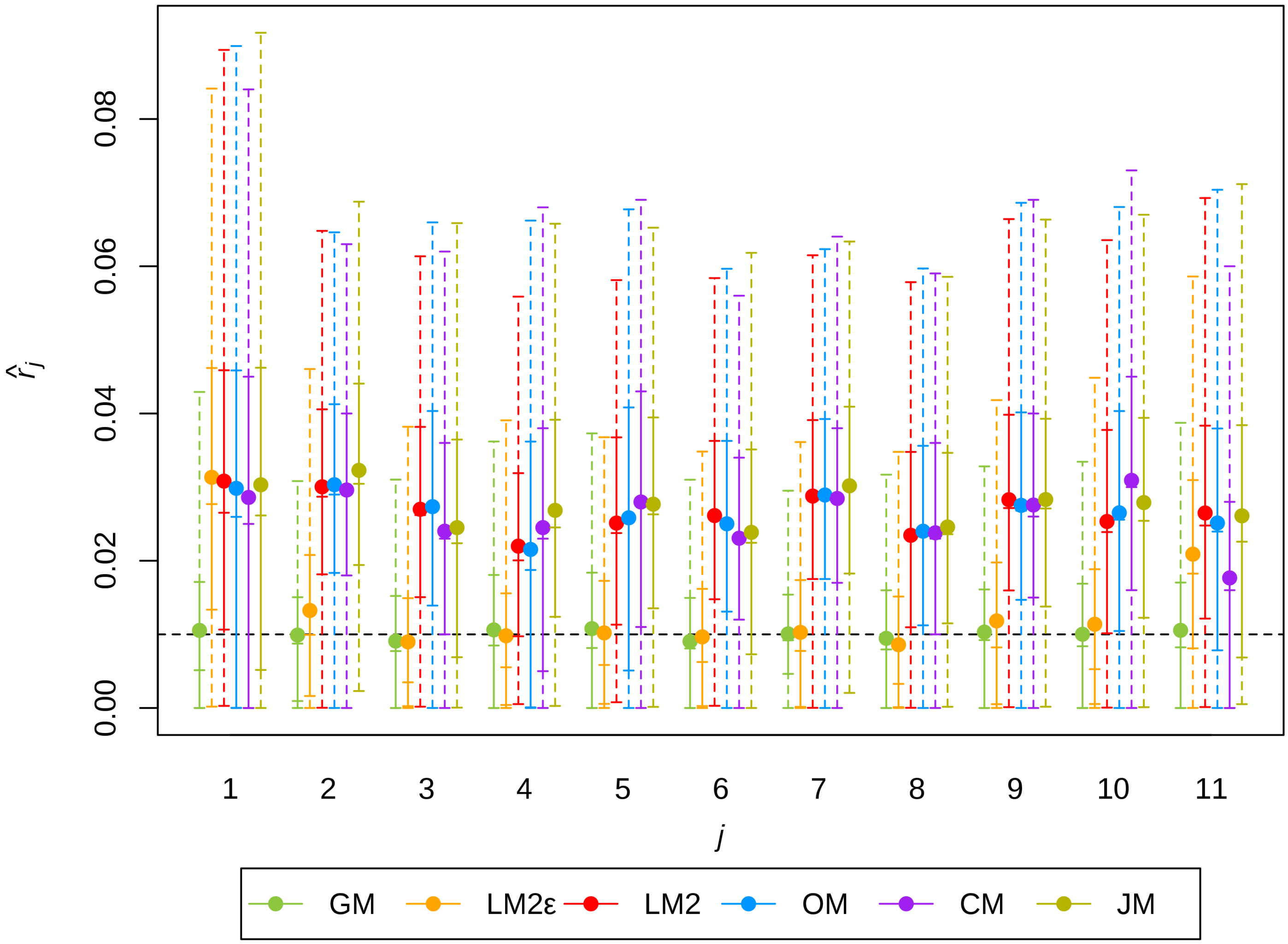

Figure S6: Distribution of the recombination fraction estimates, $\hat{r}_j$, for moderate depth and large sequencing error. The solid point represents the mean, the vertical solid line represents the interquartile range, the vertical dashed line represents the range between the 2.5^{th} and 97.5^{th} percentiles, the five horizontal solid lines represent, in ascending order, the 2.5^{th} percentile, lower quantile, median, upper quantile and 97.5^{th} percentile, and the horizontal black dotted line represents the true parameter value. j is the interval from the jth locus to the $(j+1)^{\text{th}}$ locus. The mean read depth was $\mu_{d_j} = 10$ and the sequencing error rate was $\varepsilon = 0.01$.

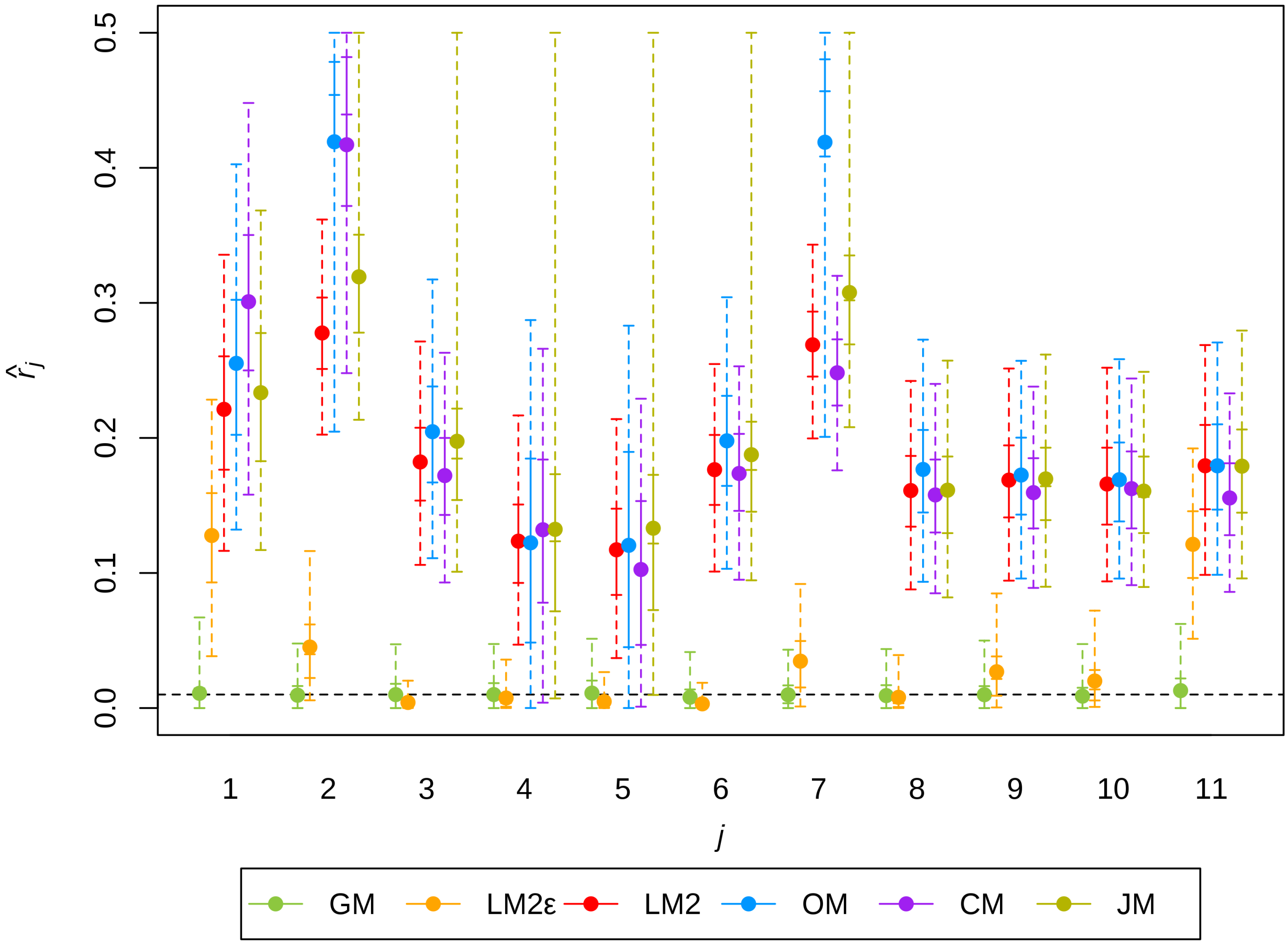

Figure S7: Distribution of the recombination fraction estimates, $\hat{r}_j$, for low depth and no sequencing error. The solid point represents the mean, the vertical solid line represents the interquartile range, the vertical dashed line represents the range between the 2.5^{th} and 97.5^{th} percentiles, the five horizontal solid lines represent, in ascending order, the 2.5^{th} percentile, lower quantile, median, upper quantile and 97.5^{th} percentile, and the horizontal black dotted line represents the true parameter value. j is the interval from the jth locus to the $(j+1)^{\text{th}}$ locus. The mean read depth was $\mu_{d_j} = 2$ and the sequencing error rate was $\varepsilon = 0$.

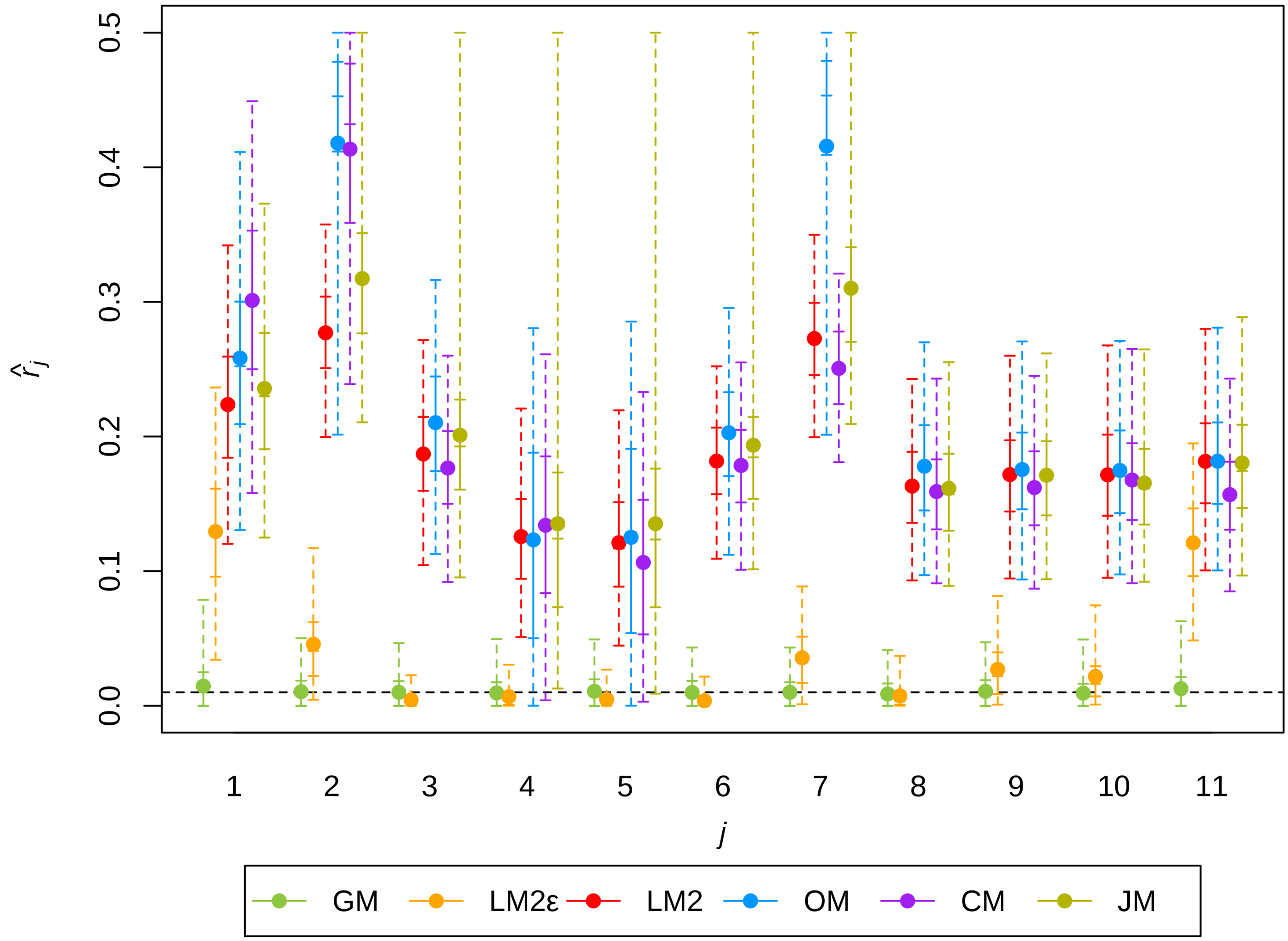

Figure S8: Distribution of the recombination fraction estimates, $\hat{r}_j$, for low depth and no small error. The solid point represents the mean, the vertical solid line represents the interquartile range, the vertical dashed line represents the range between the 2.5^{th} and 97.5^{th} percentiles, the five horizontal solid lines represent, in ascending order, the 2.5^{th} percentile, lower quantile, median, upper quantile and 97.5^{th} percentile, and the horizontal black dotted line represents the true parameter value. j is the interval from the jth locus to the $(j+1)^{\text{th}}$ locus. The mean read depth was $\mu_{d_j} = 2$ and the sequencing error rate was $\varepsilon = 0.002$.

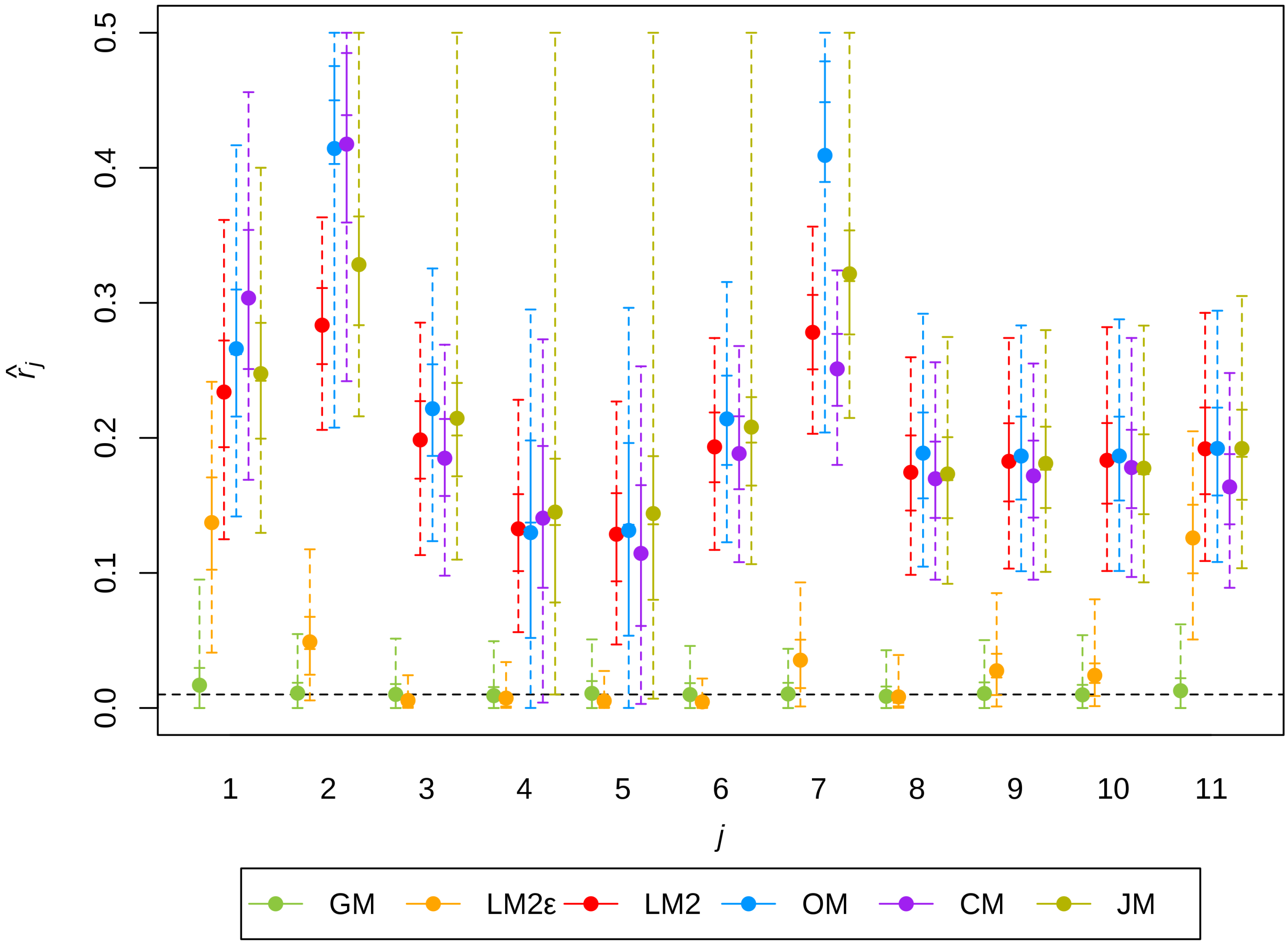

Figure S9: Distribution of the recombination fraction estimates, $\hat{r}_j$, for low depth and no large error. The solid point represents the mean, the vertical solid line represents the interquartile range, the vertical dashed line represents the range between the 2.5^{th} and 97.5^{th} percentiles, the five horizontal solid lines represent, in ascending order, the 2.5^{th} percentile, lower quantile, median, upper quantile and 97.5^{th} percentile, and the horizontal black dotted line represents the true parameter value. j is the interval from the jth locus to the $(j+1)^{\text{th}}$ locus. The mean read depth was $\mu_{d_j} = 2$ and the sequencing error rate was $\varepsilon = 0.01$.

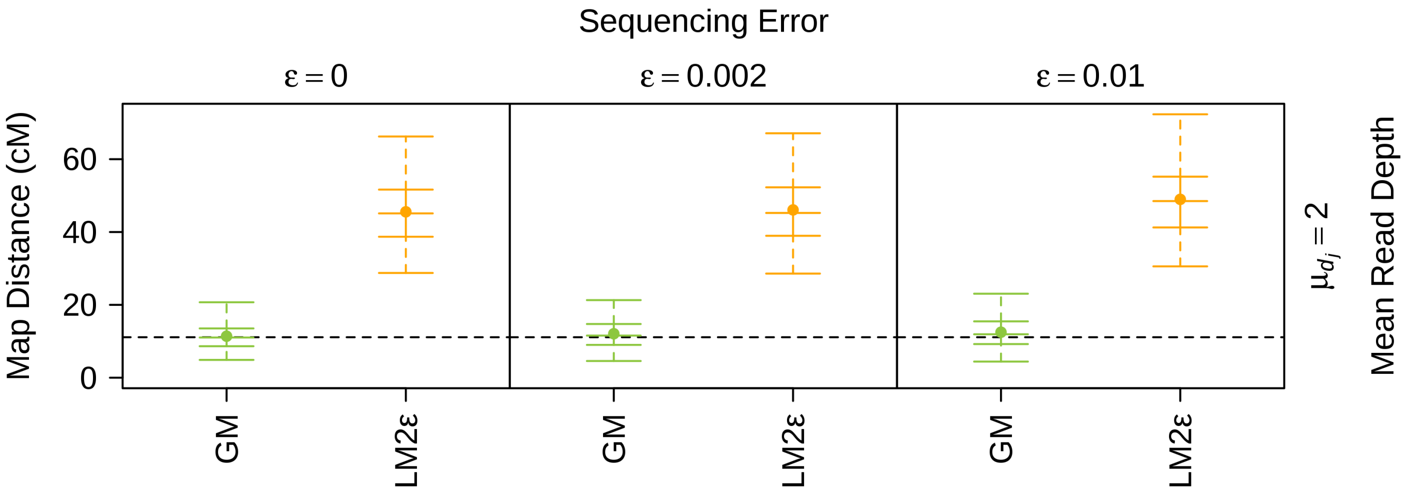

Figure S10: Distribution of the map distance estimates for GM and LM2ε at low depth. The solid point represents the mean, the vertical solid line represents the interquartile range, the vertical dashed line represents the range between the 2.5^{th} and 97.5^{th} percentiles, the five horizontal solid lines represent, in ascending order, the 2.5^{th} percentile, lower quantile, median, upper quantile and 97.5^{th} percentile, and the horizontal black dotted line represents the true parameter value. Map distances were computed using the Haldane mapping function.

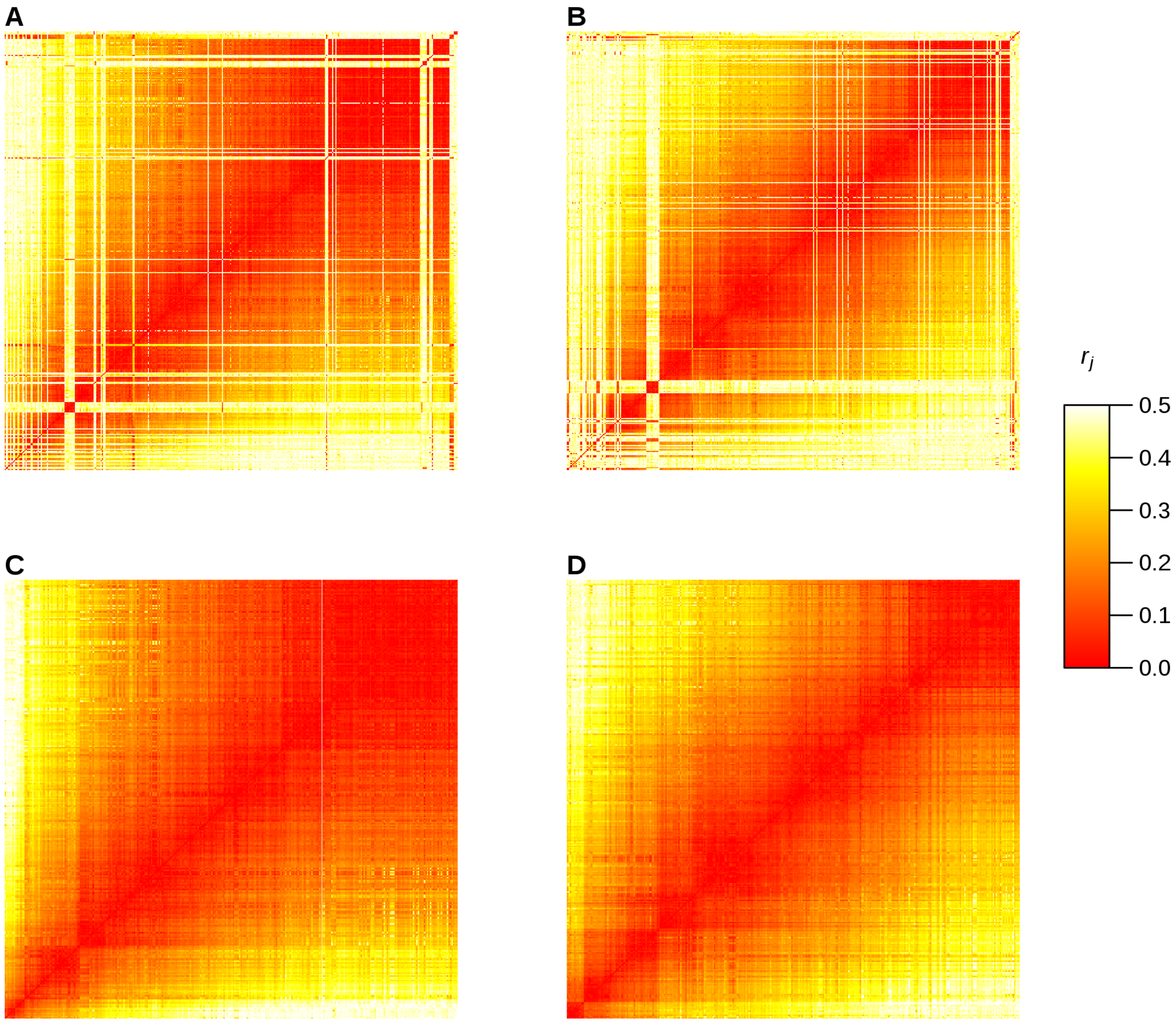

Figure S11: Heatmaps of 2-point recombination fraction estimates for SNPs on mānuka chromosome 11. The plots are for (A) PI and BI SNPs, (B) MI and BI SNPs, (C) PI and BI SNPs, where the SNPs which appear incorrectly ordered or erroneous based on (A) are removed, and (D) PI and BI SNPs, where the SNPs which appear incorrectly ordered or erroneous based on (B) are removed. Recombination fractions were estimated using GM (with $\varepsilon = 0$), where the phase was taken as the one which maximized the likelihood.

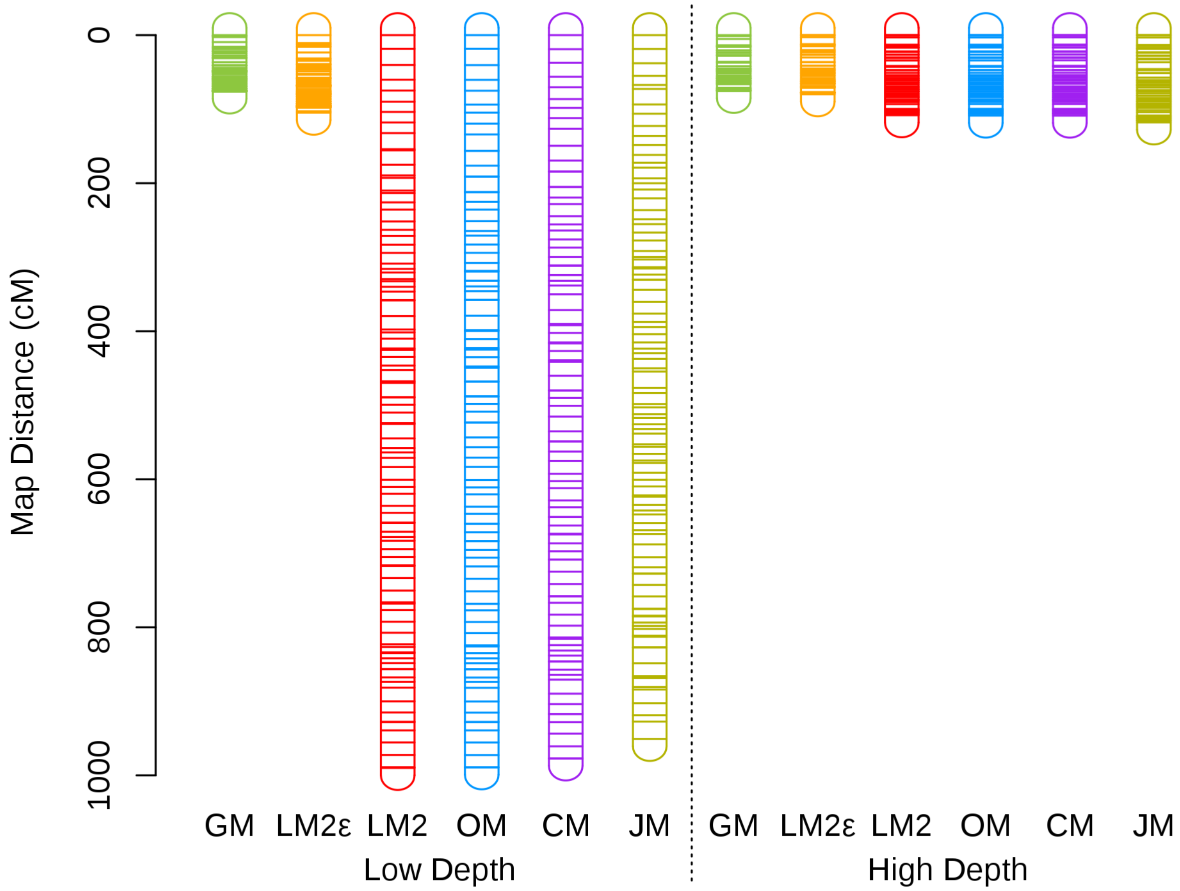

Figure S12: Linkage maps for mānuka chromosome 11. Low Depth refers to the maps produced using SNPs with a mean read depth below 6, while High Depth refers to maps produced using SNPs with less than 20% missing data after setting genotypes with a read depth below 20 to missing. Map distances are in centimorgans (cM) and were computed using the Haldane mapping function. The rounded rectangles represent the chromosomes and the horizontal lines represent the SNPs. Different set of SNPs are used in the low and high depth sets.

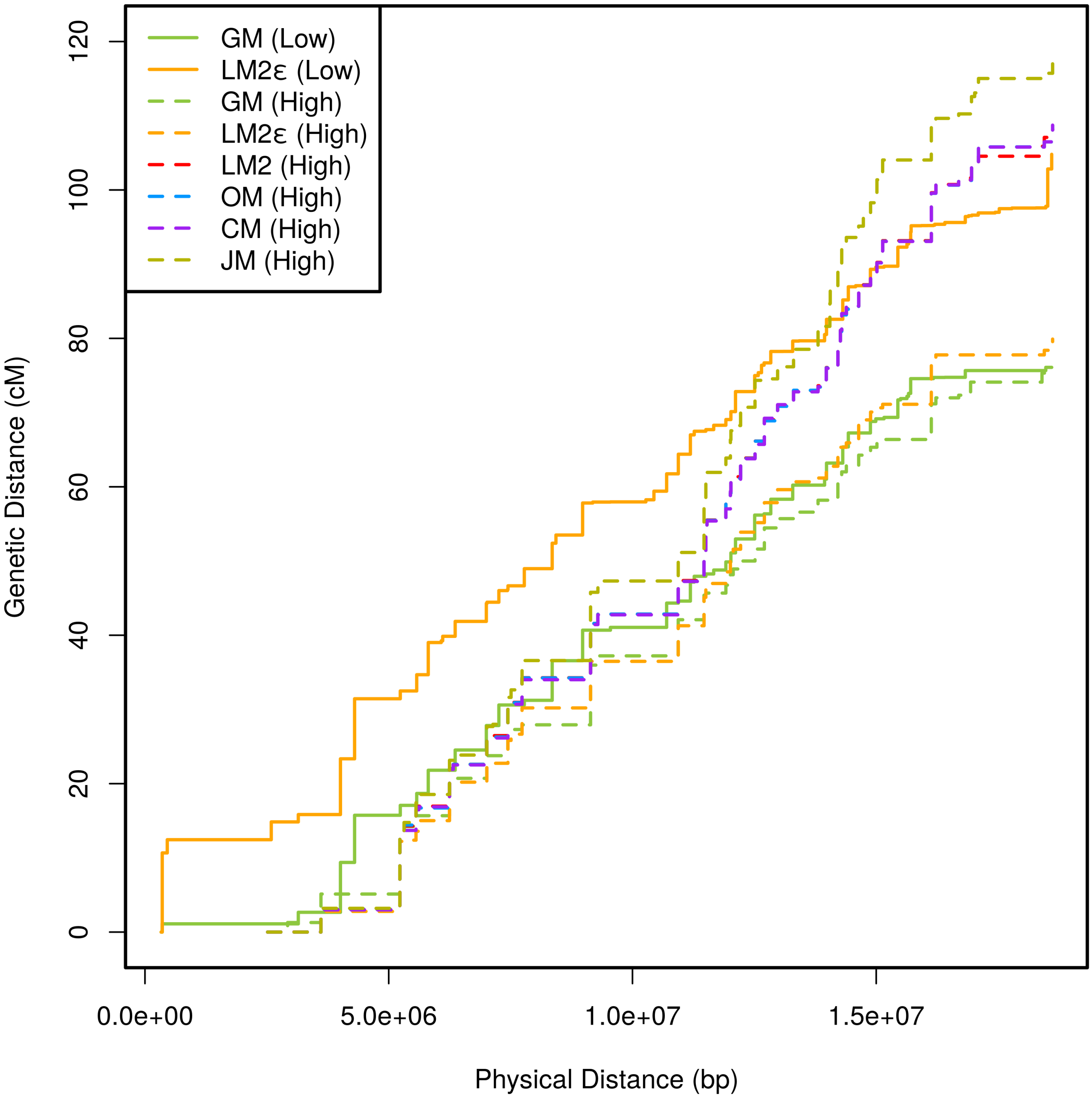

Figure S13: Plot of the cumulative genetic distance versus the physical distance for SNPs on the linkage maps given in Figure 5. The solid line represents estimation using the Low Depth SNPs while the dashed line represents estimation using the High Depth SNPs.

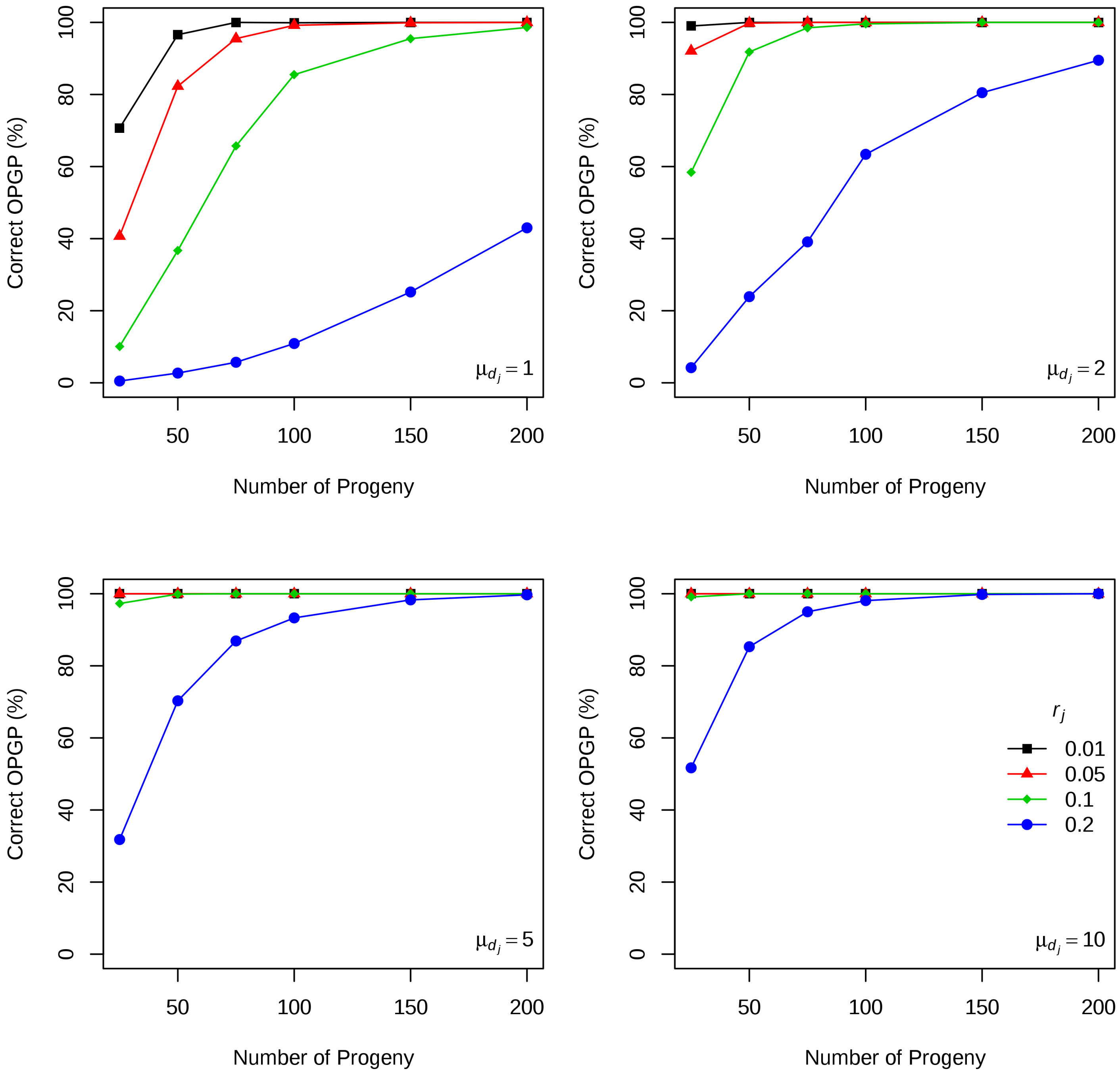

Figure S14: Simulation of ordered parental genotype pair (OPGP) inference. Percentage of data sets in which GM correctly inferred all the OPGPs for varying family sizes, true recombination fraction values and mean read depth. The parameter values used to simulate the data were $F = 1$, $M = 12$, $\varepsilon = 0.002$ and the segregation type of the loci corresponded to those given in Table 2.
